# Phthalazine as a medically versatile polyheterocyclic core with underexplored anticancer potential

**DOI:** 10.1080/14756366.2026.2717060

**Published:** 2026-09-18

**Authors:** Mikołaj Biegański, Joanna Matysiak, Monika Szeliga

**Affiliations:** ^a^ Faculty of Medicine, Medical University of Warsaw, Warsaw, Poland; ^b^ Department of Neurotoxicology, Mossakowski Medical Research Institute, Polish Academy of Sciences, Warsaw, Poland; ^c^ Department of Chemistry, University of Life Sciences in Lublin, Lublin, Poland

**Keywords:** Phthalazine derivative, cancer, inhibitor, drug discovery, clinical trials

## Abstract

Phthalazine derivatives are nitrogen-containing heterocyclic compounds that have gained considerable attention in medicinal chemistry due to their pharmacological potential. This review briefly outlines the biological properties and clinical application of five- and six-membered heterocycles. The main focus is placed on the recent advances in the development and biological evaluation of the phthalazine derivatives. A particular emphasis is put on their PARP and VEGFR inhibitory activity, which has been investigated in clinical trials for cancer treatment, ultimately leading to the market launch of some of these compounds. Furthermore, the recent studies on numerous phthalazine derivatives that demonstrate promising anticancer activity *in vitro* are presented. Finally, this review combines the previously developed synthesis strategies and possible improvements that could be implemented in the future with clinically oriented summary focused on the application of these compounds in oncology, thereby complementing current literature.

## Graphical abstract

**Figure:**
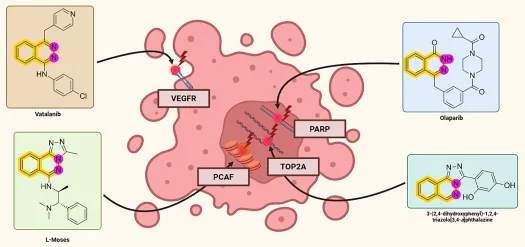

## Introduction

Cancer remains a significant public health challenge in the 21st century, accounting for nearly one in six deaths worldwide. It is estimated that by 2050, the number of new cancer diagnoses will reach 35 million^1^. Despite advancements in cancer therapy, currently available modalities still face obstacles such as limited efficacy and serious adverse effects. Therefore, there is an urgent need to develop more precise and targeted treatment strategies that would overcome the limitations of conventional therapies. The traditional drug discovery process is extremely complex, time-consuming, and expensive. Regardless of the enormous amount of work and funding involved, less than 1% of compounds will enter clinical trials, and even fewer will reach the market. Repurposing drugs already approved for use in other conditions appears to be an attractive strategy for expanding cancer treatment options^2^. Among the many classes of drug-candidate compounds, aromatic heterocycles have attracted significant attention due to their versatility, diversity of molecular targets, and the influence of structural modifications on their biological activity. Within this group, five- and six-membered rings containing oxygen and nitrogen are of particular interest. Aromatic oxygen-based heterocycles display a wide range of pharmacological properties. One of the members of this class of compounds is coumarin, a naturally occurring aromatic lactone, which has been used for many years as a core moiety in anticoagulant agents (e.g. warfarin). Furthermore, coumarin and its derivatives exhibit a broad spectrum of other properties, including anticancer activity. Several recent reports suggest that combining coumarin with other pharmacophores, such as benzimidazole, may suppress pathways engaged in cancer development or inhibit multi-drug resistance mechanisms^3^. A number of other O-heterocyclic structures are currently under investigation as potential anticancer agents, including furan derivatives^4^, chromones^5^, benzofuran derivatives^6^ and flavones^7^. Another class of compounds worth mentioning in this context are oxazoles containing both oxygen and nitrogen atoms which demonstrate promising properties as potential candidates for future selective kinase inhibitors^8^.

Another class of molecules exhibiting a wide range of biological properties are nitrogen-based heterocycles^9–12^. Some of the N-heterocyclic compounds have already undergone clinical trials and been launched on the pharmaceutical market, while the majority of the drug-candidates are currently being evaluated *in vitro*^13^ This group includes derivatives of six-membered ring pyridine, drugs developed for the treatment of multiple conditions, ranging from the Food and Drug Administration (FDA)-approved drug clopidogrel commonly used as antiplatelet agent, through omeprazole, a popular proton pump inhibitor (PPI), to varenicline, used for nicotine addiction substitutive therapy^13^. Although this group forms a notable part of modern pharmacology, it is five-membered nitrogen-containing heterocycles that should be regarded as the most developed sub-group. Derivatives of imidazole and triazole moieties have secured their place in many modern treatment guidelines, accounting for the basis of antimicrobial^14^, antifungal^15^, antiparasitic^16^ and anxiolytic^17^ therapies. Similarly, pyrazole, another five-membered nitrogen-containing heterocycle is of increasing interest in pharmacology, due to its promising antiproliferative and anticancer potential. This scaffold, which inhibits Janus kinases (JAK), has recently been introduced to the market under the trivial names ruxolitinib, crizotinib, and encorafenib, for the therapy of different cancers and inflammatory skin diseases^18^. Furthermore, these derivatives have demonstrated a satisfactory inhibitory activity against several other kinases and some oncogenes’ products. It should be noted that many popular drugs that have been available on the market for many years such as angiotensin receptor antagonists (ARBs)^19^, or phosphodiesterase type 5 (PDE5) inhibitors, used in the therapy of erectile dysfunction, contain five-membered nitrogen heterocycle ring^20^. Beside these, there are multiple other FDA-approved chemicals based on nitrogen heterocycles, including anti-inflammatory cyclooxygenases (COX) inhibitors (celecoxib, indomethacin), and tryptamine derivatives used in the treatment of migraine episodes (triptans)^21^. Quinoline, a nitrogen-containing polyheterocycle, should also be mentioned, as a lead scaffold for many well-known centuries old antimalarial agents, including quinine, chloroquine and mefloquine^22^.

Nitrogen heterocycles, due to their diverse pharmacological properties, and chemical similarity to nucleic acid bases, have been proven to be effective anticancer agents. For example, pyrimidine derivatives 5-fluorouracil and cytarabine are used as effective antimetabolic chemotherapeutics, canonically prescribed for colorectal cancer and acute myeloid leukaemia (AML), respectively^23^^,^^24^. These drugs are believed to act *via* a misincorporation to cancer cell’s DNA strand, ultimately causing deregulation of replication machinery, resulting in cell death^25^. Nitrogen heterocycle moiety is also present in structures of many novel anticancer drugs, included in action mechanism-driven group known as kinase inhibitors. Pyrimidine moiety is found in the structure of imatinib, used in haematology for the treatment of chronic myeloid leukaemia (CML)^26^, while quinazoline scaffold is incorporated in the structures of erlotinib and gefitinib, EGFR inhibitors used in the therapy of lung cancer^27^. Another molecular scaffold used in modern chemotherapy is pteridine. One of its derivatives, methotrexate, is a commonly used as cytostatic agent, working as an antagonist of folic acid metabolism^28^^,^^29^. This compound may be applied in the treatment of many neoplasms; however, it has been also found to be useful in the therapy of autoimmune disorders such as rheumatoid arthritis. Nitrogen heterocycle is also incorporated in the structure of temozolomide (TMZ), an alkylating agent used in the treatment of malignant gliomas^30^. Low molecular mass, which facilitates crossing of blood-brain barrier, and high reactivity of multiple nitrogen atoms covalently bound to carbon, results in intracellular cleavage of this compound, producing a highly unstable methyldiazonium cation, capable of transferring methyl groups to nucleic acid bases molecules^31^. Several nitrogen-containing heterocycles that have already been approved for cancer therapy are listed in Table 1.

**Table 1.** The examples of the nitrogen-containing heterocycles approved for cancer therapy. Designed by the authors using Marvin Cloud. No copyrighted material was reused.

Drug	Chemical structure	Mechanism of action	Approval status	Clinical use
Imatinib	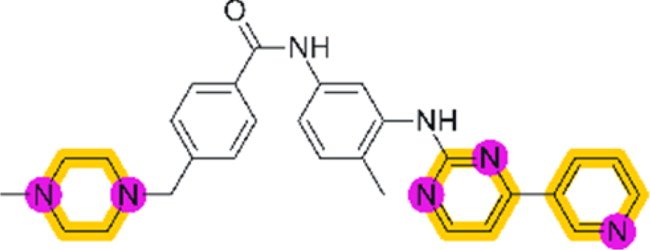	Tyrosine kinase inhibitor Confirmed activity against c-KIT (CD117), PDGFR, and BCR-ABL mutant kinase	Approved by FDA in 2001 Approved by EMA in 2001	Primarily used in chronic myeloid leukaemia (CML) therapy. Indications have been later extended, now covering also other tyrosine kinase dependent neoplasms acute lymphoblastic leukaemia (ALL), myelodysplastic and myeloproliferative syndromes (MDS/MPS), hypereosinophilic syndrome (HES), and gastrointestinal stromal tumours (GIST).
Erlotinib	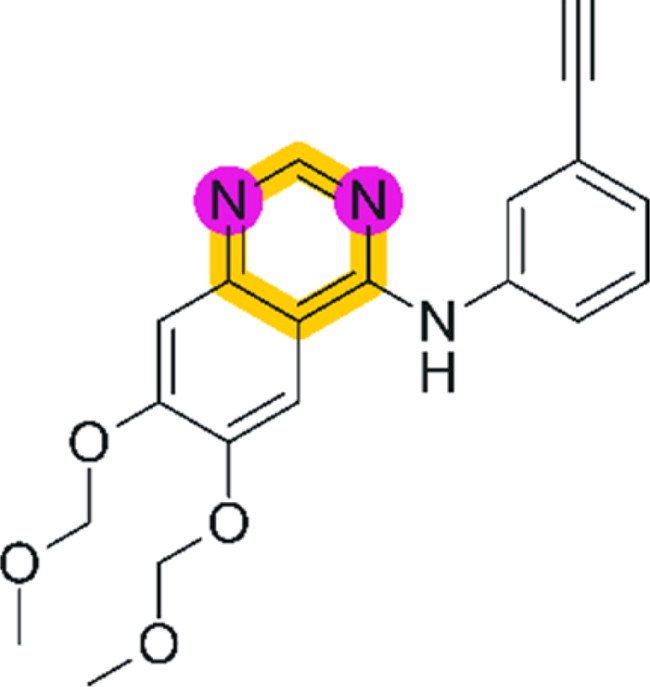	EGFR inhibitor	Approved by FDA in 2004 Approved by EMA in 2005	Used in EGFR-mutated non-small cell lung cancer (NSCLC) therapy. In combination with gemcitabine, used also in pancreatic cancer treatment.
Gefitinib	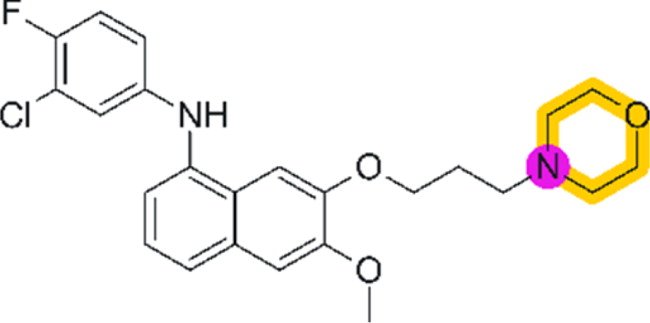	EGFR inhibitor	Approved by FDA in 2003 Approved by EMA in 2009	Used in EGFR-mutated NSCLC.
Methotrexate	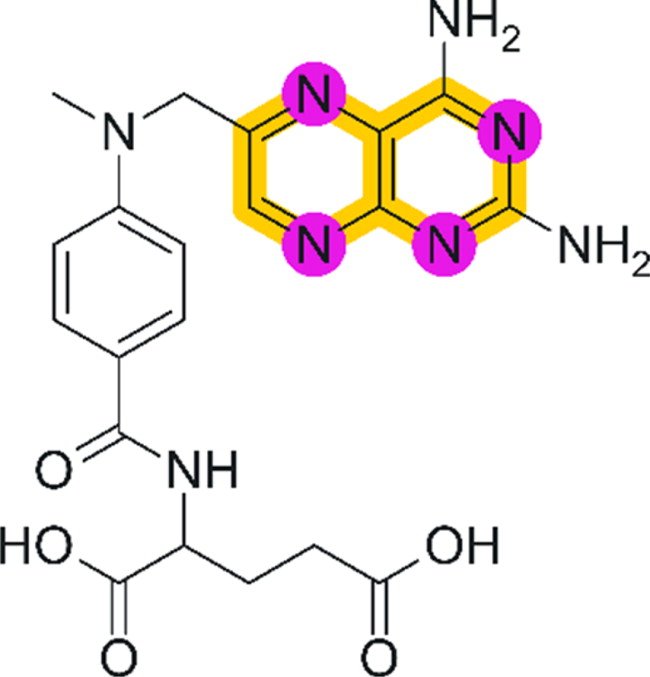	DHFR inhibitor (Folic acid inhibitor)	Approved by FDA in 1953 In Europe used since 1950s	In high doses used as chemotherapeutic in therapy of ALL, Non-Hodgkin lymphomas, choriocarcinoma, osteosarcoma, breast cancer, lung cancer subtypes, head and neck cancer, and many other. In low doses used as immunosuppressive therapeutic, indicated in many autoimmunization disorders including rheumatoid arthritis and psoriasis. Administered also in treatment of ectopic pregnancy.
Temozolomide	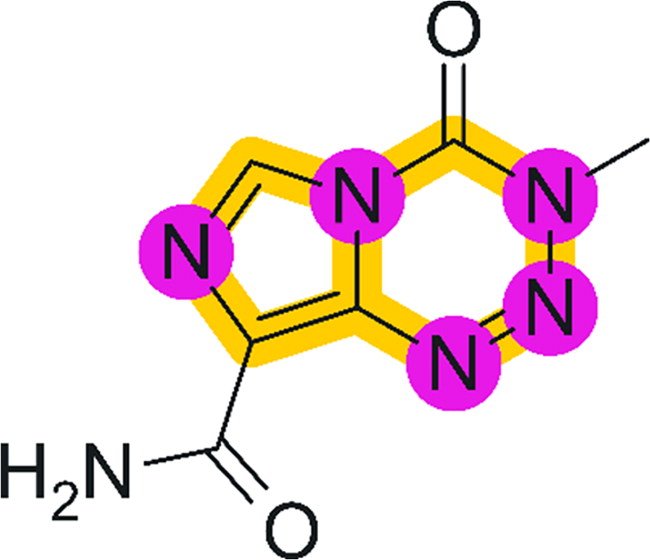	DNA alkylating agent	Approved by FDA in 1999 Approved by EMA in 1999	Used in the therapy of malignant brain lesions, including anaplastic astrocytoma (AA), and glioblastoma (GBM).

While many pyridazine, imidazole, triazole, tryptamine and quinazoline derivatives described previously have been proven to be effective in therapy of multiple diseases, derivatives of many other anticancer nitrogen-containing heterocycles are currently in development. Among these, tetrazoles^32^, N-heterocyclic carbenes^33^, and phthalazines^34^ are considered promising anticancer agents.

Tetrazole derivatives, an example of nitrogen-containing heterocycles with 4 nitrogen atoms incorporated into five-membered ring, are characterised by extraordinary chemical properties, due to the existence in several tautomeric forms of nitrogen-rich ring. This feature broadens the spectrum of possible biological activity, resulting in both antimicrobial and anticancer properties^32^. While tetrazole derivatives represent a conservative approach of modifying known structure seeking new activities, N-heterocyclic carbenes constitute completely new pharmacological approach, utilising the unique chemical properties of carbenes, molecules containing a non-ionized carbon atom with paired electrons. Carbometallated derivatives of N-heterocycles were tested against multiple cancer models, including cell lines and patient-derived organoids, proving these compounds to be effective and cancer-selective^33^.

**Figure 1.**
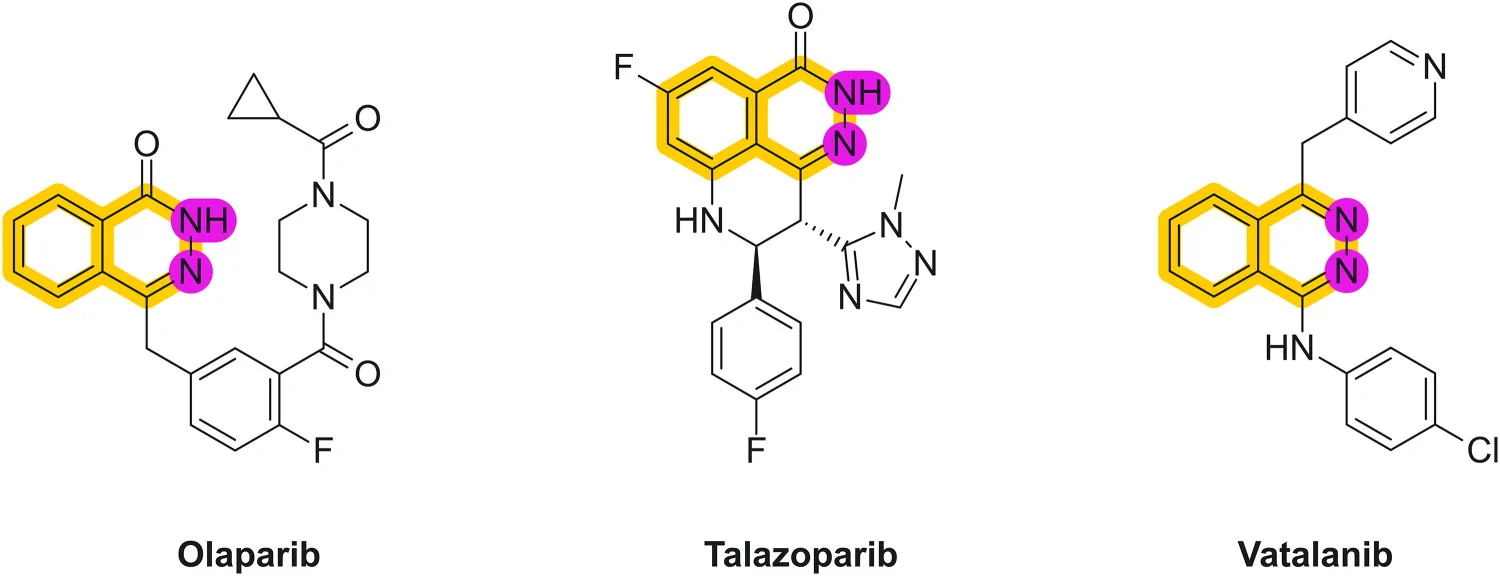
Phthalazine-based anti-cancer drugs (phthalazine moiety highlighted). Designed by the authors using Marvin Cloud. No copyrighted material was reused.

Some phthalazine-based drugs have already been clinically tested as potential anticancer agents (Figure 1). Furthermore, a considerable number of derivatives have been examined in many *in vitro* models, revealing potential antimicrobial, anti-inflammatory, antihypertensive, and anticancer properties of these compounds^35^, that were summarised in several literature reviews published recently^34^^,^^36^^,^^37^. However, these reviews are mainly focused on the synthetic methodologies used for the construction of the phthalazine cores and/or present the broad spectrum of their pharmacological properties in a wide context. This review combines data from clinical trials, pre-clinical reports, and most recent synthetic possibilities to obtain the concise overview of phthalazine derivatives in clinical oncology and cancer research. Thus, we delve into the molecular mechanisms underlying anticancer activity of the specific derivatives and present how the modifications of the core structure influence this activity. Furthermore, we introduce the phthalazine-based drugs currently approved in the clinics, as well as updates from clinical trials of drug candidates. Given the growing interest in drug repurposing, we shed light on the phthalazine-based drugs already approved for non-oncology indications. We also propose potential future directions that would enable broader use of this promising group of compounds in medicinal chemistry.^36^

## Literature selection methods

The literature included in this review was identified through searches of PubMed and Scopus databases. The following keywords were used to search the databases: “phthalazine derivative”, “phthalazinone”, “nitrogen heterocycles”. These were combined with terms such as “cancer”, “anticancer”, “glioblastoma”, “breast cancer”, “colorectal cancer”, “clinical trial”, “synthesis”, “molecular docking”, “target”, “SAR”, “repurposing”, “*in vitro*”, “phase I”, “phase II”, “phase III”. Only original research articles, reviews, and clinical trial reports written in English, published in peer-review journals, were subjected to further analysis. Initially retrieved preclinical studies were prioritised according to their translational maturity (*in silico*, *in vitro*, *in vivo*) and comprehensiveness of drug characterisation. For *in silico* studies, we sought later-published experimental validation, or external confirmation of simulations results. Selected clinical trial reports and repurposing studies enrolling patients were prioritised according to their role in an introduction of particular compound to pharmaceutical market (or its abandonment), together with a chronological order of publication. Studies on synthesis were prioritised based on their usefulness for drug production. Synthesis methods were presented in an order of increasing complexity of entry compounds. This review covers relevant studies published between 1981 and 2026, with particular emphasis on the most recent reports published from 2024 to 2026.

## Clinically evaluated phthalazine based drugs and drug candidates

### Phthalazinone derivatives as PARP inhibitors

**Figure 2.**
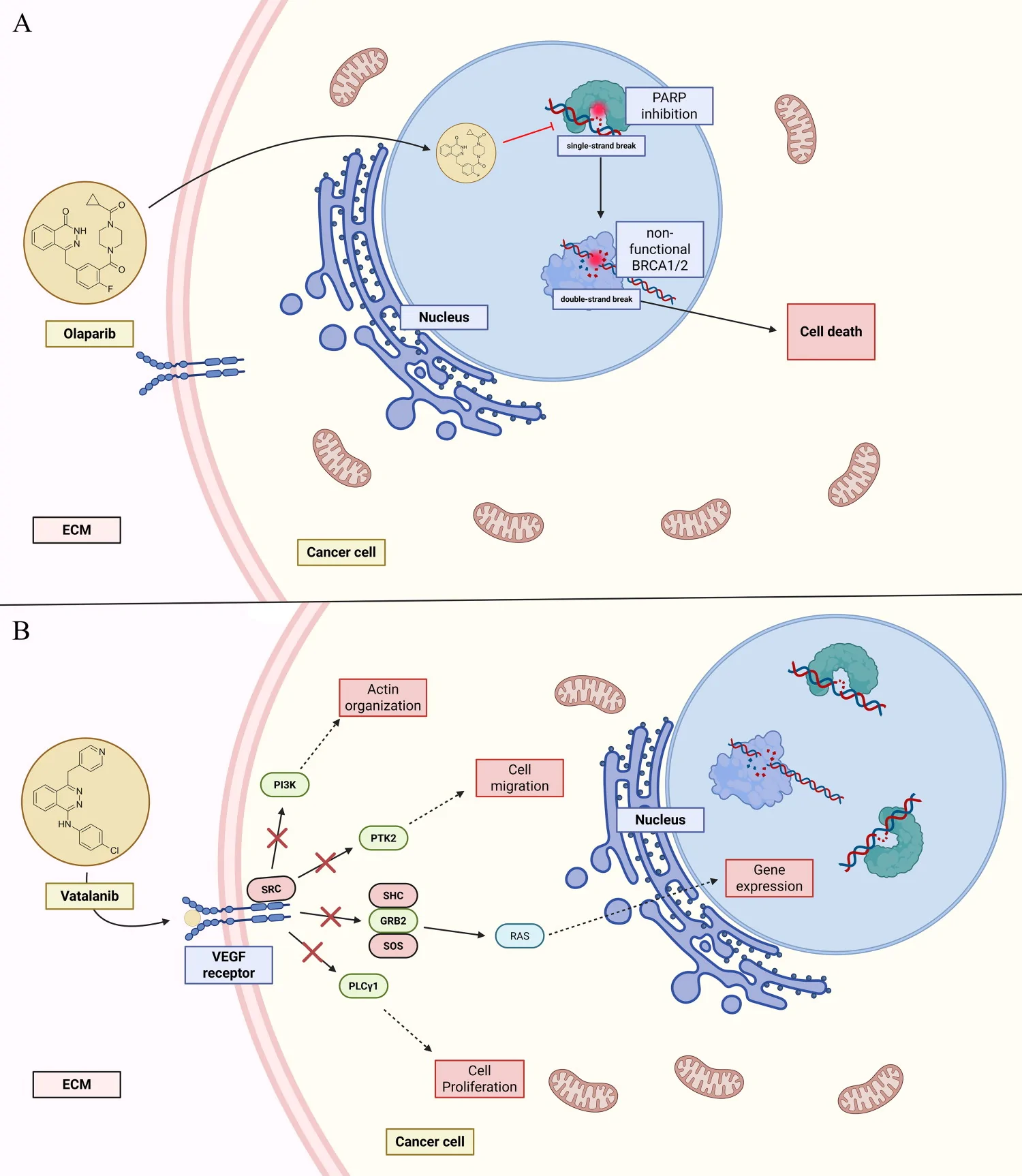
Mechanism of action of phthalazine-based PARP and VEGFR inhibitors. Designed by the authors using BioRender®. No copyrighted material was reused.

The most significant milestone in the development of phthalazinones (amidic derivative of phthalazine) as potential anticancer agentstook place in 2008, when Rottenberg et al.^38^ published the first report of pre-clinical trial of a drug, known at that time as AZD2281 (4-[[3-[4-(cyclopropanecarbonyl)piperazine-1-carbonyl]-4-fluorophenyl]methyl]-2*H*-phthalazin-1-one), later renamed as olaparib (Figure 1). This novel molecule targeted poly(ADP-ribose) polymerase 1 (PARP1), an enzyme responsible for the maintenance of DNA strands integrity and repair of single-strand breaks, that is crucial for survival of cancer cells with disrupted homologous recombination (HR) often caused by *BRCA1*/*BRCA2* mutation (Figure 2A). In this study, olaparib was found to significantly elongate overall survival and relapse time in mice with *Brca1* and *p53* genes knockout. Moreover, the compound was characterised by excellent therapeutic index, without any dose-limiting toxicity found in tumour-bearing mice. The authors concluded that olaparib was a promising agent in the therapy of triple negative breast cancer (TNBC) associated with *BRCA1* mutation^38^. One year later, phase I clinical trial NCT00516373 report was published, proving olaparib effectiveness and safety^39^. The report confirmed PARP1 inhibition in olaparib treated patients, across the enrolled group of 60 patients who had ovarian, breast or prostate cancer refractory to standard therapies, among whom 22 carried a mutation in *BRCA1* or *BRCA2*. It should be mentioned that the treatment response (defined as partial or complete response according to RECIST criteria) was observed only in patients with HR dysregulation. A subsequent phase II pre-registration clinical trial involving 265 patients with platinum-sensitive serous ovarian cancer confirmed antitumour activity of olaparib, showing significantly prolonged median progression-free survival (mPFS) in the study group. Notably, olaparib showed the highest activity in *BRCA*-mutated patients^40^. Based on these findings, olaparib was ultimately introduced to the market as FDA and European Medicines Agency-approved drug in 2014. In subsequent large-scale trials olaparib has been proven to extend mPFS in patients with *BRCA1*-mutated ovarian and breast cancer. OlympiAD phase III clinical trial (NCT02000622) summarised in 2017, revealed that olaparib monotherapy prolonged mPFS by 2.8 months compared with standard therapy in patients with *HER2*-negative metastatic breast cancer and a germline *BRCA* mutation, and risk of disease progression or death was found to be 42% lower than for standard protocol^41^. Results from phase III SOLO1 clinical trial (NCT01844986), published in 2018, demonstrated that in patients with ovarian cancer carrying *BRCA1/2* mutations treatment with olaparib was associated with a 70% lower risk of disease progression or death compared with placebo^42^. Simultaneously run phase III clinical trial SOLO2 (NCT01874353) showed that maintenance therapy olaparib in patients with platinum-sensitive relapsed ovarian cancer (PSROC) with a *BRCA1/2* mutation extended mPFS by 13.6 months (19.1 vs 5.5 in placebo group)^43^.^43^ The other phase III trial, SOLO3 (NCT02282020), demonstrated that in patients with *BRCA1/2*-mutated PSROC overall survival (OS) was similar in olaparib-treated and non-platinum chemotherapy groups (34.9 vs 32.9 months, respectively). Favourable OS (37.9 vs 28.8 months) was observed in patients who had received two previous lines of chemotherapy.^44^. Importantly, a report on phase III POLO trial (NCT02184195), published in 2019, revealed that in patients with *BRCA1/2*-mutated metastatic pancreas cancer, mPFS was longer in the olaparib group than in the placebo group (7.4 vs 3.8 months), with no significant difference in OS (18.9 vs 19.1 months)^45^.

Olaparib is the first phthalazinone derivative to reach market. Satisfactory outcomes of patients treated with olaparib and deeper understanding of *BRCA1/2* mutation role in cancer cell resulted in an extension of its indications, now including not only *BRCA1/2*-mutated prostate and pancreatic cancer, but also maintenance therapy of *BRCA1/2-*mutated ovarian cancer relapse^46^. Success of olaparib propelled the development of other PARP inhibitors, one of which is also a phthalazinone derivative. Talazoparib (Figure 1), initially known as BMN 673 or MDV3800 was primarily tested *in vitro* in 2013, revealing an up to 200-fold greater PARP inhibition activity when compared to existing inhibitors, with PARP1 IC_50_ (Half Maximal Inhibitory Concentration) as low as 0.57 nM^47^. This study has also demonstrated that oral administration of talazoparib to rats with xenografted breast cancer cells carrying *Brca* or *Pten* mutations elicited remarkable antitumour activity, leading to almost complete disappearance of xenografts after 30-days therapy. These promising results led to phase I clinical trial NCT01286987, in which a total of 110 patients with advanced solid tumours were treated with constant or increasing dose of talazoparib According to the report on this trial published in 2018, the minimal dose of talazoparib required to achieve sustained PARP inhibition is 0.60 mg/day. In the study cohort, the objective response rate (ORR) in patients with *BRCA* mutation-associated breast and ovarian cancer was 50% and 42% respectively^48^. Results of this study contributed to FDA approval of talazoparib in 2018. A subsequent phase II clinical trial ABRAZO (NCT02034916) demonstrated higher ORR to talazoparib in patients with advanced breast cancer and *BRCA* mutation previously treated with at least 3 different courses of chemotherapy (excluding platinum compounds) compared to patients previously treated with platinum compounds (37% *vs* 21%)^49^. Phase III EMBRACA clinical trial (NCT01945775) conducted on a randomised cohort of 431 patients with *BRCA*-mutated HER2-negative advanced breast cancer, published in 2020, confirmed substantial benefits of talazoparib therapy, eliciting significantly higher ORR (62.2% *vs* 27.2%) and prolonged mPFS (8.6 months *vs* 5.6 months), compared to standard therapy^50^. However, it should be noted that in none of the clinical trials conducted so far has treatment with talazoparib been directly associated with any significant changes in median overall survival (mOS)^51^. Furthermore, mOS extending effect was observed for olaparib only in one study (SOLO3) in specific patients group, while no statistically significant OS benefit was demonstrated in other phase III trials.

It should be emphasised that both olaparib and talazoparib molecules contain phthalazin-1(2H)-one core moiety (an amidic derivative of phthalazine), instead of a bare phthalazine double-ring. Molecular docking studies suggest that this moiety seems to be important for olaparib molecule binding to PARP1 protein, forming hydrogen bonds with arginine and tyrosine residues in the nicotinamide-binding (NI) site, and strengthening the interaction *via* π-π stacking with nearby tyrosine residues^52^. One should bear in mind that these interactions are simulated to be carried out mainly by the carbonyl group of the amide moiety, absent in bare phthalazine moiety, so these interactions may not be present for other compounds based on this scaffold. This fact suggests an existence of other mechanisms facilitating an anticancer activity of phthalazine derivatives.

### Phthalazine derivatives as tyrosine kinase inhibitors

First reports suggesting a potential anticancer activity of phthalazine derivatives date back to 2000, with reports published by Wood et al.^53^, introducing a putative inhibition of vascular endothelial growth factor receptor (VEGFR) by PTK787/ZK222584 (Figure 2B). This drug, chemically classified as a pyridinyl phthalazine derivative, was found to inhibit an activity of other receptor kinases, including platelet-derived growth factor receptor (PDGFR). Further development of this compound proved the antiproliferative effect of this drug in combination with irradiation, both in human umbilical vein endothelial cells (HUVECs) and human colon carcinoma cells (SW480) xenografts in nude mice^54^. After pre-clinical testing, the drug renamed as vatalanib (N-(4-chlorophenyl)-4-(pyridin-4-ylmethyl)phthalazin-1-amine) (Figure 1) entered clinical trials as a potential therapeutic for multiple neoplasms. Phase I clinical trials revealed vatalanib to be well-tolerated as a monotherapy agent (with maximum-tolerated oral dose of 750 mg twice a day)^55^, and in combination with cetuximab in the treatment of advanced solid tumours^56^. In 2006, a phase I clinical trial (NCT00358163) was initiated to assess the safety and efficacy of combined therapy comprising vatalanib with paclitaxel. However, as of July 2026, no report on this trial had been published. Phase II clinical trials investigating vatalanib monotherapy for gastrointestinal stromal tumour (GIST) (NCT00117299)^57^, non-small cell lung cancer (NCT00160043)^58^, myelodysplastic syndromes (NCT00072475)^59^, and phase I trial for glioblastoma (NCT00385853)^60^ did not find any significant improvement in participants’ condition, compared with standard therapies. Subsequent randomised phase III clinical trials in colorectal cancer, CONFIRM-1 (NCT00056459, previously untreated patients)^61^ and CONFIRM-2 (NCT00056446, patients with disease progression on previous 5-fluorouracil/irinotecan therapy)^62^, revealed that an addition of vatalanib to standard FOLFOX4 treatment protocol (5-fluorouracil, leucovorin, oxaliplatin) did not extend mOS, while mPFS was only slightly extended exclusively in pre-treated (CONFIRM-2) cohort. Clinical trials remarkably contributing to the market introduction of phthalazine-based drugs, or an abandonment of further development of aforementioned molecules are listed in Table 2.

**Table 2.** Clinical trials leading to FDA approval of phthalazine-based anticancer drugs (or discontinuation of their development).

Drug	Clinical trial no.	Target neoplasm	Phase	Status	Trial primary endpoint(s)	Outcome	Ref.
Olaparib	NCT00516373	Breast cancer, prostate cancer	I	Completed	Safety Tolerability Toxicity DLT MTD	The drug achieved PARP inhibition by more than 90%, compared with the value at baseline. Study revealed olaparib to demonstrate satisfactory antitumour activity in BRCA-mutated individuals (secondary endpoint).	^39^
Olaparib	NCT00753545	Platinum-sensitive serous ovarian cancer	II	Completed	PFS	The drug has been proven active against serous ovarian cancer. The treatment was found associated with prolonged mPFS, with no significant changes to mOS (secondary endpoint).	^40^
Olaparib	NCT02000622 (OlympiAD trial)	Breast cancer	III	Completed	PFS	Olaparib monotherapy was found associated with significantly longer mPFS with higher response rate, compared with standard therapy.	^41^
Olaparib	NCT01844986 (SOLO1 trial)	Ovarian cancer, peritoneal cancer, fallopian-tube cancer	III	Active, not recruiting	PFS	Maintenance therapy with olaparib was found associated with 70% lower risk of death or disease progression.	^42^
Olaparib	NCT01874353 (SOLO2 trial)	Platinum-sensitive *BRCA1/2*-mutated ovarian cancer	III	Active, not recruiting	PFS	Maintenance therapy was found associated with significantly increased mPFS in comparison to placebo group (19.1 vs 5.5 months).	^43^
Olaparib	NCT02282020 (SOLO3 trial)	Platinum-sensitive *BRCA1/2*-mutated ovarian cancer previously treated with at least 2 lines of chemotherapy	III	Completed	ORR	Patients with two previous chemotherapy lines showed remarkably higher ORR (72.2% vs 51.4%) compared to non-platinum chemotherapy treatment. The same group demonstrated higher mPFS and mOS (16.4 vs 9.0 months, and 37.9 vs 28.8 months respectively).	^44^
Olaparib	NCT02184195 (POLO trial)	*BRCA1/2*-mutated pancreatic cancer	III	Completed	PFS	Olaparib therapy showed significantly extended mPFS (7.4 vs 3.8 months) when compared with placebo group.	^45^
Talazoparib	NCT01286987	Advanced solid tumours	I	Completed	ORR PFS DOR SD MTD	Study confirmed satisfactory response rates in BRCA-mutated breast cancer (50%) and ovarian cancer (42%).	^48^
Talazoparib	NCT02034916 (ABRAZO trial)	Advanced breast cancer	II	Completed	ORR	Talazoparib therapy was found associated with high (37%) ORR in patients pretreated with at least 3 different chemotherapy courses (excluding platinum). ORR observed in patients with platinum-pretreated tumours was lower (21%).	^49^
Talazoparib	NCT01945775 (EMBRACA)	BRCA-mutated, HER2-negative advanced breast cancer	III	Completed	PFS	Talazoparib therapy was found associated with mPFS (8.6 months vs 5.6 months) and ORR (62.2% vs 27.2%, secondary endpoint) significantly higher, compared to standard therapy. No significant changes in mOS (secondary endpoint) were found.	^50^ ^,^ ^51^
Vatalanib	–	Advanced solid tumours	I	Completed	Safety Pharmacokinetics MTD	The study revealed a maximum-tolerated oral dose of 750mg BID. A biologically active dose was established as >1000mg daily.	^55^
Vatalanib + cetuximab	–	Advanced solid tumours	I	Completed	Safety and tolerability	Combined therapy with cetuximab and vatalanib was well tolerated by patients. One out of 14 patients showe a partial response, while 7 patients (50%) had stable disease for at least 2 months.	^56^
Vatalanib + paclitaxel	NCT00358163	Metastatic non-hematologic malignancies	I	Terminated	Safety Pharmacokinetics	No report published.	^-^
Vatalanib	NCT00117299	Imatinib-resistant gastrointestinal stromal tumour (GIST)	II	Completed	ORR	Vatalanib demonstrated limited antitumour activity against imatinib-resistant GISTs. Vatalanib was found to be well-tolerated by GIST patients (secondary endpoint).	^57^
Vatalanib	NCT00160043	Non-small cell lung cancer	II	Completed	ORR	Vatalanib demonstrated potential benefits in tumor size reduction, disease control rate and survival, with moderate toxicity profile.	^58^
Vatalanib	NCT00072475	Myelodysplastic syndromes	II	Completed	ORR Time to AML transformation	Vatalanib was found to improve blood counts in small sub-group of patients. Clinical applicability was assessed as limited, due to severe side effects.	^59^
Vatalanib + FOLFOX	NCT00056459	Colorectal cancer (untreated)	III	Completed	PFS	No significant changes in primary endpoint.	^61^
Vatalanib + FOLFOX	NCT00056446	Colorectal cancer (previously treated)	III	Completed	PFS	No significant changes in primary endpoint.	^62^

DLT – Dose-Limiting Toxicity; MTD – Maximum Tolerated Dose; PFS – Progression-Free Survival; OS – Overall Survival; ORR – Objective Response Rate; SD – Stable Disease; AML – Acute Myeloid Leukaemia.

## Anticancer drug candidates assessed in preclinical studies

**Figure 3.**
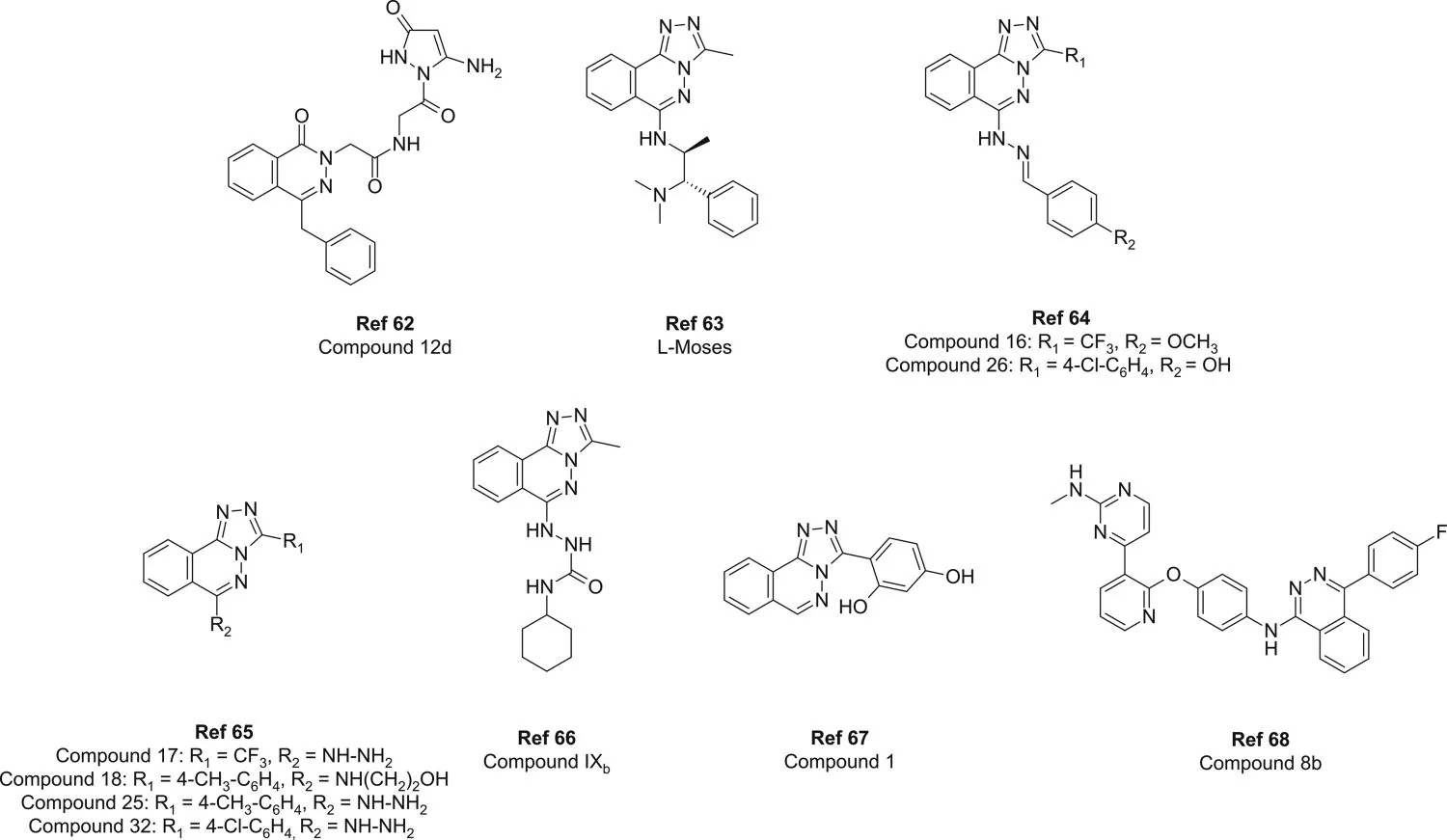
Phthalazine derivatives showing anti-cancer activity *in vitro*. Designed by the authors using Marvin Cloud. No copyrighted material was reused.

Phthalazinone derivatives (Table 3) were investigated as potential inhibitors of other receptor kinases, such as epidermal growth factor receptor (EGFR). Compounds proposed by Emam et al.^63^, structurally based on phthalazinone scaffold substituted with long side chain containing acetamidoacetamide moieties, have shown EGFR inhibition activity, with IC_50_ measured for the most active compound (Compound 12d, Figure 3) equal to 21.4 nM, value remarkably lower than for an established EGFR inhibitor erlotinib. These findings were supported by molecular docking study, yielding a satisfactory calculated binding energy (ΔG) of −18.4 kcal/mol, and demonstrated on a model of breast cancer cell lines MCF7, MCF10A and MDA-MB-231. While the cytotoxic effect of the studied compounds was observed for all the examined cell lines, the latter one additionally demonstrated elevated apoptosis rate upon 48 h treatment with studied compounds.

**Table 3.** Phthalazine derivatives investigated as anticancer agents at the preclinical stage.

Compounds group	Reference(s)	Target(s)	Model(s)	Assay type(s)	Best potency value (IC_50_)	Translational maturity
Phthalazinone derivatives (12d and similar)	62	EGFR	Breast cancer (MCF-7, MDA-MB-231, MCF-10A)	EGFR inhibition Cytotoxicity (MTT) Apoptosis (Annexin V-FITC)	12d IC_50_: 21.4 ± 0.67 nM 12d IC_50_: 0.57 ± 0.09 against MDA-MB-231 12d: Total apoptosis rate for MDA-MB-231 42.5%	*In silico* (docking study) and *in vitro* studies
1,2,4-triazolo[3,4-*a*]phthalazine derivatives (L-Moses and similar)	62-65	PCAF	Hepatocellular carcinoma (HePG2), Breast cancer (MCF-7), Prostate cancer (PC3), colorectal carcinoma (HCT116)	PCAF inhibition Cytotoxicity (MTT) Apoptosis (Annexin V-FITC)	17 IC_50_ (ref 50): 5.31 ± 0.23 μM 17 IC_50_ (ref 50): 3.06 ± 0.2 μM against HePG2 Total apoptosis rate for HePG2 26.51%	*In silico* (docking study) and *in vitro* studies
1,2,4-triazolo[3,4-*a*]phthalazine derivatives (IXb and similar)	66, 67	TOP2A	Hepatocellular carcinoma (HePG2), Breast cancer (MCF-7), colorectal carcinoma (HCT-116), Glioblastoma (U87MG, LN229, PDCL)	Topoisomerase II inhibition DNA intercalation assay Cytotoxicity (MTT) Apoptosis (Annexin V-FITC)	IXb IC_50_ (ref 51): 7.38 ± 0.62** **µM IXb IC_50_ (ref 51): 27.16 ± 1.2 µM IXb IC_50_ (ref 51): 3.81 ± 0.2 µM against MCF-7 IXb (ref 51): Total apoptosis rate for HePG2 19.35%	*In silico* (docking and MD study) and *in vitro* studies
Pyridinyl-Pyrimidine Phthalazines derivatives (8b and similar)	68	Aurora kinases	Cervical cancer (HeLa), colorectal carcinoma (COLO 205 mice xenograft)	Aurora A/B inhibition H3S10/DAPI flow cytometry analysis of cell cycle COLO 205 xenograft evaluation of pharmacokinetics	8b IC_50_: 41 nM (Aurora-A), 15 nM (Aurora-B) 8b: Concentration of 0.313 µM reduced phosphorylation of H3S10 and increased percentage of cells with >4N DNA content 8b: Significant inhibition of pH3S10 was maintained after 3h and 6h after treatment. Compound 8b was found to be highly bound to mouse plasma proteins (>99.9% bound)	*In silico* (docking study), *in vitro* and *in vivo* studies

Other derivatives of 1,2,4-triazolo[3,4-*a*]phthalazine have been investigated as putative histone acetyltransferase inhibitors. The discovery of the first of these compounds, later referred to as L-Moses, opened the door to selective inhibition of bromodomain-containing proteins. This compound selectively inhibits human P300/CBP-associated factor (PCAF) histone acetyltransferase, known also as KAT2B ((1*S*,2*S*)-N1,N1-dimethyl-N2–(3-methyl-[1,2,4]triazolo[3,4-*a*]phthalazin-6-yl)-1-phenylpropane-1,2-diamine) (L-Moses, Figure 3)^64^. Further development of the 1,2,4-triazolo[3,4-*a*]phthalazine led to discovery of other, more potent bromodomain inhibitors^65^^,^^66^, with anticancer and apoptosis-inducing properties confirmed in *in vitro* setting. The most potent derivatives are presented in Figure 3. SAR (Structure–Activity Relationship**)** studies indicated that these structures are prone to further modifications, and the bromodomain-binding potency can be further upgraded. Molecular docking studies for these compounds suggested that planar structure of phthalazine moiety is important for binding to PCAF bromodomain’s lysine binding site, fitting sterically well into a narrow binding pocket. However, direct interactions with amino acid residues responsible for structure and gating of PCAF’s binding site Ans1436 and Glu1389 were mainly mediated by phthalazine’s nitrogen-containing ring substituents, which would suggest that the primary role of phthalazine core is to anchor the molecule in the binding site. Interestingly, similar 1,2,4-triazolo[3,4-*a*]phthalazine derivatives were later investigated as putative DNA intercalators, again due to their planar and rigid structure. Study by Sakr et al.^67^ investigated multiple derivatives with different substituents, concluding that these compounds should be considered both DNA intercalators and topoisomerase IIα (TOP2A) inhibitors. The compounds synthesised in this study decreased viability of several cancer cell lines, including hepatocellular carcinoma HepG2, breast cancer MCF7, and colorectal cancer HCT-116 cells. The topoisomerase IIα inhibitory activity was confirmed by the direct enzyme inhibition assay and the topoisomerase II-mediated DNA cleavage measurement, yielding the IC_50_ value for the most potent derivative IXb (*N*-cyclohexyl-2-(3-methyl-[1,2,4]triazolo[3,4-*a*]phthalazin-6-yl)-hydrazine-1-carboxamide) as low as 7.38 ± 0.62** **µM and satisfactory formation of linear DNA after treatment with 2.5 µM of IXb. Furthermore, this compound induced apoptosis in HepG2 cells and increased the percentage of cells in G2/M and pre-G1 phases. Molecular docking studies suggested that several studied compounds are able to stably interact with DNA strand *via* π-π stackings formed between planar phthalazine moiety and aromatic ring of nucleic acid bases, with ΔG as low as −51.09 kcal/mol for derivative X (ΔG for the most biologically active derivative IXb was estimated at −49.23 kcal/mol). Molecular dynamics (MD) simulation performed in a separate study suggested that a similar derivative, compound 1 (3–(2,4-dihydroxyphenyl)-1,2,4-triazolo[3,4-a]phthalazine, Figure 3) tend to localise between nucleic acid bases aromatic ring, with the distance in range of π-π stacking interaction^68^. However, it should be noted that authors observed an instability of the dsDNA (double-stranded DNA) strands immediately after contact with studied molecule, which resulted in high root mean square deviation (RMSD) value for the system. These *in silico* findings were supported by biological evaluation showing the significant reduction of viability and clonogenicity of LN229, U87MG and patient-derived glioblastoma cell line (PDCL) treated with compound 1. Yet, it should be emphasised that the presented mechanistic observations are based mainly on *in silico* computational methods prone to be biased by hardware limitations and were not tested directly in experimental framework.

Pyridinyl-pyrimidine substituted phthalazine derivatives have been investigated as molecules targeting non-receptor serine/threonine kinases, such as Aurora kinase. These molecules were observed to inhibit both Aurora A and Aurora B kinases, inducing polyploidy in human cervical cancer HeLa cell line. Moreover, these compounds were characterised by favourable pharmacokinetic properties in comparison to anthranilamide derivatives, including oral bioavailability of one of derivatives equal to 61% (compound 8b, Figure 3)^69^. The ability of compound 8b to inhibit Aurora B activity was confirmed in a pharmacodynamic assay measuring phosphorylation of the Aurora B substrate histone H3^68^.

The data presented above indicate a significant association between an inclusion of phthalazine scaffold in a drug candidate and an anticancer activity mediated *via* multiple mechanisms. The primary observation, supported by previously presented docking and MD studies, is that the presence of this moiety may augment or even determine the interaction with the narrow binding pockets of specific proteins, such as aforementioned PCAF. However, emerging SAR evidence, together with broad range of experimentally proven molecular targets, highlights the importance of substituents, which, under several conditions, may play the role of key moiety by facilitating or enhancing the inhibitory activity. Indeed, SAR analysis presented by Emam et al.^63^ demonstrates strong dependence of the EGFR inhibitory activity on the structure of terminal substituent, unequivocally favouring five-membered rings. This observation deserves particular attention, as the structure of the compounds presented by these authors renders the terminal substituent the least sterically hindered moiety, making it especially interesting as a potential interactor of proteins with complex domain architectures such as tyrosine kinases. Moreover, docking studies performed for the phthalazine-dsDNA-Topo II system highlighted the importance of distal substituents as moieties responsible for enhancing the intercalation of phthalazine into nucleic acid molecule *via* multiple modes of interaction with amino acid residues of main protein^67^. On the other hand, SAR data obtained in the course of the development of phthalazine-based inhibitors of proteins with predominantly single catalytic domains such as PCAF, suggest a negative correlation of substituent size with activity^65^^,^^66^. This may be caused by a proximity of the substituent to the phthalazine ring in previously published studies, which concluded that the blockage of active site may be the key mode of action of these compounds. On the other hand, the anticancer activity of 1,2,4-triazolo[3,4-a]phthalazine derivatives was significantly enhanced by the introduction of an electron-donating moiety, such as a hydroxyl group at the *para* position of terminal phenyl ring of the benzylidenehydrazone substituent, suggesting that both the electronic properties and hydrogen-bonding capability of the substituents may contribute to the anticancer effect. Nevertheless, the planar structure of the phthalazine moiety constitutes the pharmacologically useful chemical individual that may be employed either as a main pharmacophore, or as an anchor in a conjugate with other potent pharmacophores.

## Phthalazine-based drugs repurposing and biological activities beyond anticancer effect

**Figure 4.**
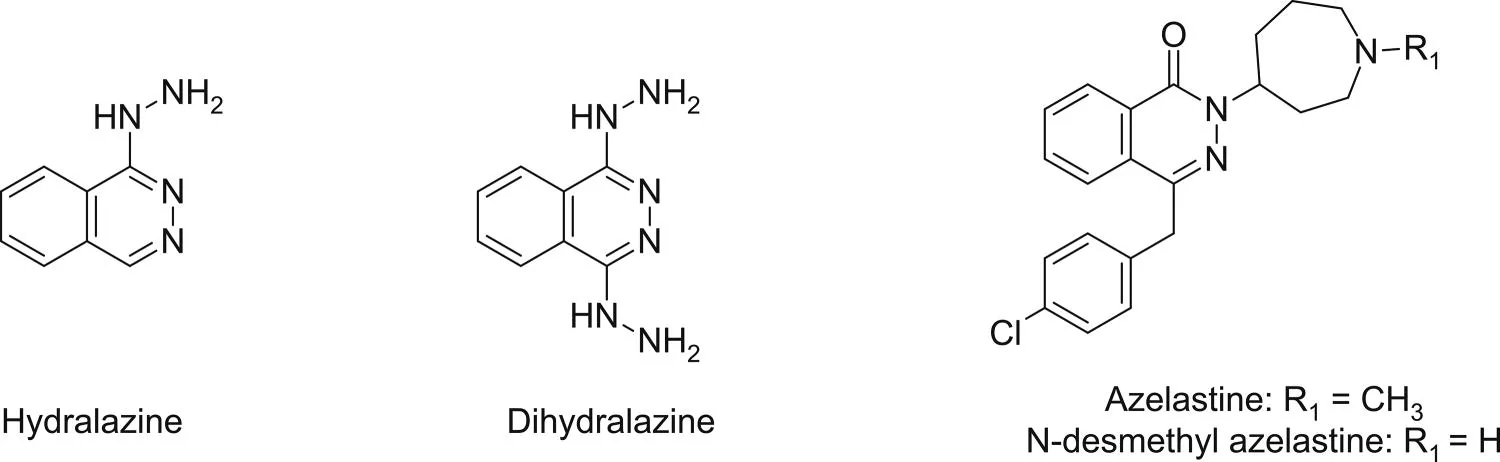
Phthalazine derivatives demonstrating antihypertensive or anti-allergic activities. Designed by the authors using Marvin Cloud. No copyrighted material was reused.

The earliest and most recognised application of phthalazine derivatives is the treatment of arterial hypertension. Hydralazine (Figure 4), and its derivative dihydralazine, have been used as antihypertensive vasodilators since the 1950s, with hydralazine receiving the official FDA approval in 1953^70^. The precise mechanism of action of these molecules remains unknown and seems to be more complex than simple nitric oxide donors, relying rather on an active blocking of inositol triphosphate-induced Ca^2+^ release from sarcoplasmic reticulum, than on an intramolecular cleavage^71^. Numerous side effects have been associated with hydralazine administration in the course of large-scale clinical analyses, indicating that persistent popularity of these drugs should be attributed mainly to the historical context of their usage^70^. While these facts underscore the necessity for further analyses of the mechanisms of action of these drugs, recent reports suggest that hydralazine administration effects may go beyond vasodilation effect.

The authors of a study published in 2025 reported that long-term hydralazine use in patients with arterial hypertension may be associated with a dose-dependent decrease in risk of haematologic neoplasms^72^. Additional evidence suggests that combining hydralazine with other repurposed drugs, including all-trans retinoic acid, may inhibit cancer cell proliferation^73^. *In vitro* studies focused on repurposing of hydralazine have documented its  ability to block the catalytic activity of apurinic/apyrimidinic endonuclease 1 (APE1), a central enzyme in the base excision repair (BER) pathway engaged in DNA repair machinery^74^^,^^75^. These reports suggest an unveiled potential of molecules containing hydrazinophthalazine core and underscore the rationale for further exploration of their properties. Nevertheless, it should be noted that available clinical trials reports are mainly effect-driven, while the elucidation of the mechanism underlying hydralazine anticancer activity is currently at the early stage of investigation. Therefore, as of 2026, hydralazine is not officially accepted as an anticancer agent.

Aside from the antihypertensive activity, selected phthalazine derivatives are currently used as antiallergic medications, commonly prescribed for seasonal allergic rhinitis. Azelastine (Figure 4), containing phthalazinone moiety substituted with N-methyl-aza-cycloheptane ring and methyl-*para-*chlorophenyl moiety, was first synthesised in 1971, being investigated for its antiallergic activity in clinical trials^76–78^. Following confirmation of its effectiveness and safety, the compound was registered as FDA-approved drug in 1996. Subsequently, large-scale randomised clinical trials, such as NCT00561717 (GEYSER clinical trial), was conducted to investigate the effects of azelastine in greater detail; however, the results of these trials have not yet been published. Nonetheless, azelastine remains a popular antiallergic drug, reaching the peak sales during the pollen season, and numerous recent reports suggest that this compound is as a promising repurposing candidate.

Azelastine, together with the main product of its liver metabolism N-desmethyl azelastine (Figure 4), were investigated as putative agents for selected human neoplasms. These compounds demonstrated toxicity against TNBC cells *in vitro*. Anticancer effect of azelastine was linked to the inhibition of ADP-ribosylation factor 1 (ARF1), which is overexpressed in TNBC and associated with poor prognosis^79^. In another study treatment of HeLa cervical cancers cells with azelastine resulted in an activation of caspase 3/7-mediated apoptosis pathway, inactivation of Bcl-2 protein, LC3-mediated autophagy induction, activation of cathepsins, induction of DNA damage, and generation of reactive oxygen species (ROS)^80^. Capitalising the momentum of recent events, azelastine was also considered an antiviral agent^81^. Initial computational analyses identified azelastine among the large set of 2700 clinically tested drugs as a potential anti-SARS-CoV-2 agent. This finding was further investigated experimentally on Vero E6 cells stably overexpressing human TMPRSS2 protein and ACE2 receptor, crucial for entry of SARS-CoV-2 into the cell. In Vero E6 cells infected with mutated spike protein, azelastine demonstrated preventive and therapeutic antiviral effects, with EC_50_ (Effective Concentration 50%) values of 3.7 and 4 μM. This effect was only slightly decreased by the introduction of other mutations into infecting agent, suggesting azelastine to be potentially active against multiple SARS-CoV-2 sub-types. Cell lines-based observation was further extended to tissue experiments, which confirmed the therapeutic effect of azelastine nasal spray on nasal tissue previously infected with SARS-CoV-2 virus, decreasing the viral RNA copy number to <0.01% in 72h after infection, compared to the untreated group. Azelastine potential as a ligand for ACE2 was further confirmed both by *in silico* and experimental approaches. The docking study of azelastine to ACE2 receptor showed the molecule to sterically well into ACE2’s binding pocket and interacting Lys353 by forming a hydrogen bond. Further investigation conducted on ACE2-overexpressing HEK293T (ACE^h^) cells revealed azelastine to exhibit SARS-CoV-2 viropexis inhibition in a concentration-dependent matter^82^. Furthermore, azelastine has been investigated as antimicrobial agent, active especially against chlamydia. This activity, previously observed in genital infection model based on HeLa cervical cancer cells infected with *Chlamydia trachomatis*, was previously linked to an antagonism of histamine 1 receptor (H1R). In an ocular infection model based on human conjunctival epithelial cells infected with ocular *Chlamydia trachomatis* strain or *Chlamydia muridarum*, an antichlamydial effect was observed for both strains, being not associated with incubation with different H1R antagonists or agonists, which suggests an H1R-independent mechanism^83^. Nonetheless, as of today, the exact molecular mode of action of azelastine as antichlamydial agent has not been elucidated.

**Figure 5.**
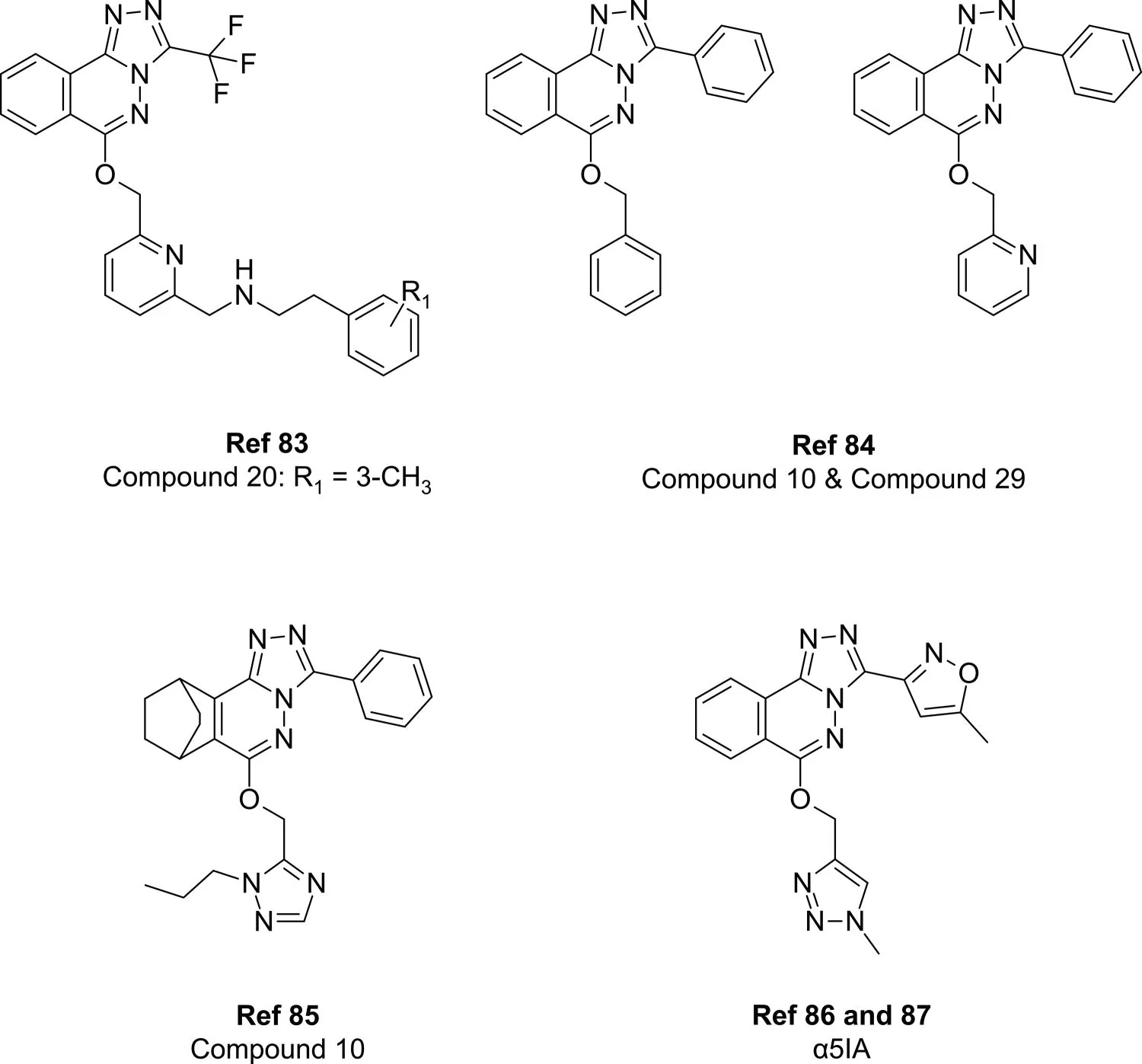
Phthalazine based compounds acting as voltage-gated calcium channels or GABAergic receptors inhibitors. Designed by the authors using Marvin Cloud. No copyrighted material was reused.

The other class of compounds demonstrating versatile activity are 1,2,4-triazolo[3,4-*a*]phthalazine derivatives mentioned previously in the section 2. The putative targets investigated for these compounds include voltage-gated calcium channels and GABA-ergic receptors. Phthalazine scaffolds substituted with long nitrogen-containing side chains, fused with triazole moiety substituted with isoxazole ring were examined as high-affinity blockers of α_2_δ-1 subunit of voltage-gated calcium channels [65]. SAR study identified nitrogen-containing group to be the main contributor to binding affinity, showing massive changes in binding IC_50_ measured for compounds containing different side chains. Affinities for the final synthesis products were measured to be as low as 15 nM, rendering further development of these compounds reasonable (Compound 20, Figure 5)^84^. More substituted phthalazine cores, containing isoxazole and triazole moieties, were developed as antagonists of α1-, α2-, α3- and α5-containing GABA_A_ receptors, being candidates for a non-benzodiazepine anxiolytic agents^85^^,^^86^. The most active compounds reported in the literature to date are presented in Figure 5. Interestingly, an intensively investigated member of this group, known as α5IA (3–(5-methylisoxazol-3-yl)-6-[(1-methyl-1,2,3-triazol-4-yl)methyloxy]-1,2,4-triazolo[3,4-a]phthalazine) was reported capable of causing the prodromal network dysfunction in hippocampus *via* altering the α5GABA_A_ receptors activity modulating hippocampal ripples, therefore mimicking the prodromal period of an early onset of Alzheimer’s disease^87^^,^^88^.

**Figure 6.**
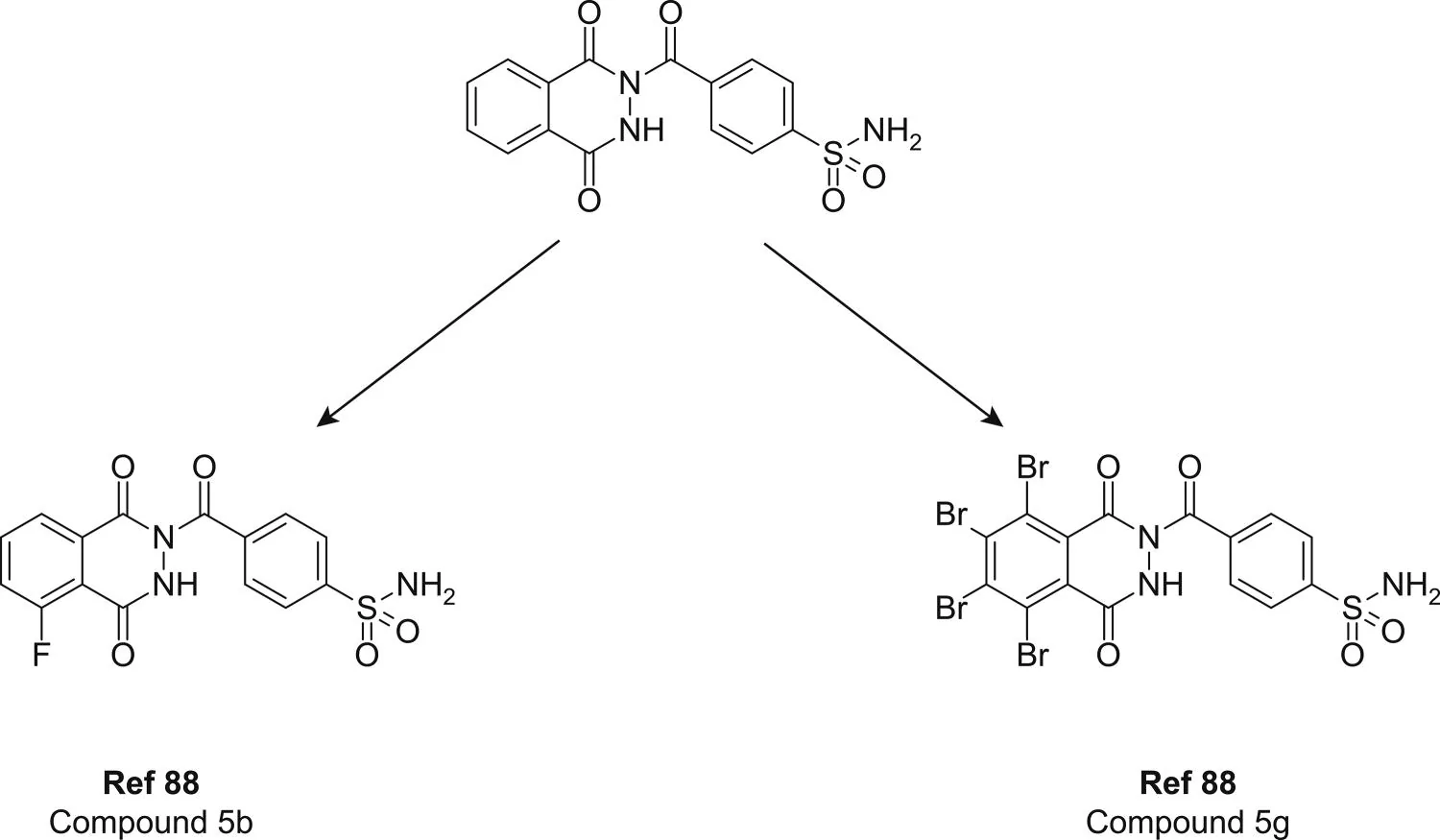
Phthalazine-based benzenesulfonamides acting as aldolase reductase (AR) and carbonic anhydrase (CA) inhibitors. Designed by the authors using Marvin Cloud. No copyrighted material was reused.

While various phthalazine derivatives mentioned above are characterised by an existence of multiple substituents, one specific sub-group of these compounds is characterised by broad reactivity and promising pharmacological properties. N-substituted phthalazine sulphonamides constitute the combination of very reactive phthalazine scaffold with pharmacologically widely used hydrophilic sulphonamide moiety^89^. The docking simulations preformed for some of these compounds showed the phthalazine scaffold to reach the deep region of the binding pocket. However, it should be noted that this specific binding mode may be augmented by a nitro group present on a phthalazine ring in these compounds, that was simulated to mediate an interaction with deep binding pocket residues Tyr48 and His110^89^. This type of interaction was observed between phthalazine N-sulphonamides and an aldolase reductase (AR), identified as one of the molecular targets for these compounds (Figure 6). SAR studies revealed these compounds to act both as competitive and non-competitive AR inhibitors, with interaction type and strength dependent on the N- substituents of the phthalazine moiety^89^. These compounds demonstrated a satisfactory binding affinity, reaching an inhibition IC_50_ around 97 nM for the most active derivative, which means it was more potent than epalrestat, the only clinically approved AR inhibitor. Importantly, the majority of these compounds does not violate the Lipinski’s rules, therefore their pharmacological profile may be considered promising.

N-substituted phthalazine sulphonamides are characterised by broad reactivity that may be reflected in a range of targets going well beyond the AR, such as carbonic anhydrase (CA) or acetylcholinesterase (AChE). Interestingly, the inhibitory activity observed for these compounds were significantly higher than those obtained for AR inhibitors, reaching IC_50_ values for CA and AChE inhibition as low as 10.14 and 15.07 nM. This observation may be associated with differences in calculated binding modes for these compounds, which would suggest entering the binding pocket in a reverse orientation compared to AR inhibitors. CA/AChE inhibitors were simulated to reach the bottom of the binding pocket with their sulphonamide moiety, which was the main interactor of the receptor’s residues. This observation may be attributed to different ligand profiles of these proteins, promoting interactions of the binding pocket deep residues with hydrophilic sulphonamide group^90^.

## Challenges limiting market introduction of phthalazine-based compounds

Despite the promising *in vitro* properties, only a very limited number of phthalazine derivatives have successfully advanced to subsequent stages of research. This may be caused by several significant obstacles encountered during the process of development. Primarily, the planar structure and a high π-π interactions forming propensity of phthalazine core moiety renders it a highly biologically active structure. Although in oncological pharmacology this property may be beneficial, and worth further investigation, it may also contribute to the promiscuous activity of phthalazine derivatives, leading to a limited target selectivity and increased off-target effects. It is worth mentioning that, although the majority of the presented studies were investigating phthalazines as interactors of specific targets, several presented derivatives possess a considerable binding affinity to other molecules^53^^,^^63^^,^^68^^,^^69^^,^^89^. This observation complicates the development process, forcing the SAR-based interventions to rely on pharmacophores decreasing cross-reactivity, instead of increasing target inhibition. Moreover, the history of vatalanib development clearly shows that significant off-target activity observed during preclinical *in vitro* studies may compromise clinical efficacy and contribute to the subsequent discontinuation of drug development program. Additionally, high reactivity of core moiety may contribute to overall toxicity of the compound, which would cause severe side effects if translated to clinical trials. Computational predictions performed for phthalazine derivatives suggest that these compounds may possess both mutagenic and toxic properties, with CYP-mediated hepatotoxicity being of particular concern. Moreover, some of them should also be considered persistent chemicals dangerous to the environment^68^. Above mentioned properties significantly reduce the interest in further development of these compounds, as it increases the risk of future discontinuation of development. Finally, the aromatic core present in these compounds may negatively influence their aqueous solubility due to increased hydrophobicity. This trait introduces a remarkable uncertainty regarding future development, forcing researchers and developers to merge these scaffolds with more hydrophilic moieties, therefore hindering both production process and potential clinical use.

## Synthesis of lead compounds

Many methods for the synthesis of the basic phthalazine skeleton have been developed and they are often used to obtain more complex framework, for example, those fused with other heterocyclic rings. Several easily available starting reagents, such as phthalic acid and its derivatives, benzoic acid derivatives, or other o-dicarbonylaryl analogues, can be converted to 1,4-disubstituted derivatives. In the reactions with hydrazine, these substrates undergo [4 + 2] two component cyclocondensations to form the phthalazine scaffold^91^^,^^92^. Some researchers have developed routes involving aromatic hydrocarbons and the AlCl_3_ catalysed Friedel–Crafts reactions as one of the initial steps in the phthalazine synthesis. The reactions employing the retro Diels–Alder processes or transformations of other heterocyclic systems into the desired structures have also been reported. Currently, many strategies for obtaining phthalazines involve multicomponent reactions (MCRs)^92^, based on the metal catalysed [3 + 2 + 1] three-component reaction^92^. Some of them meet the requirements of green chemistry^36^.

### Reaction of dicarbonylaryl compounds

**Figure 7.**
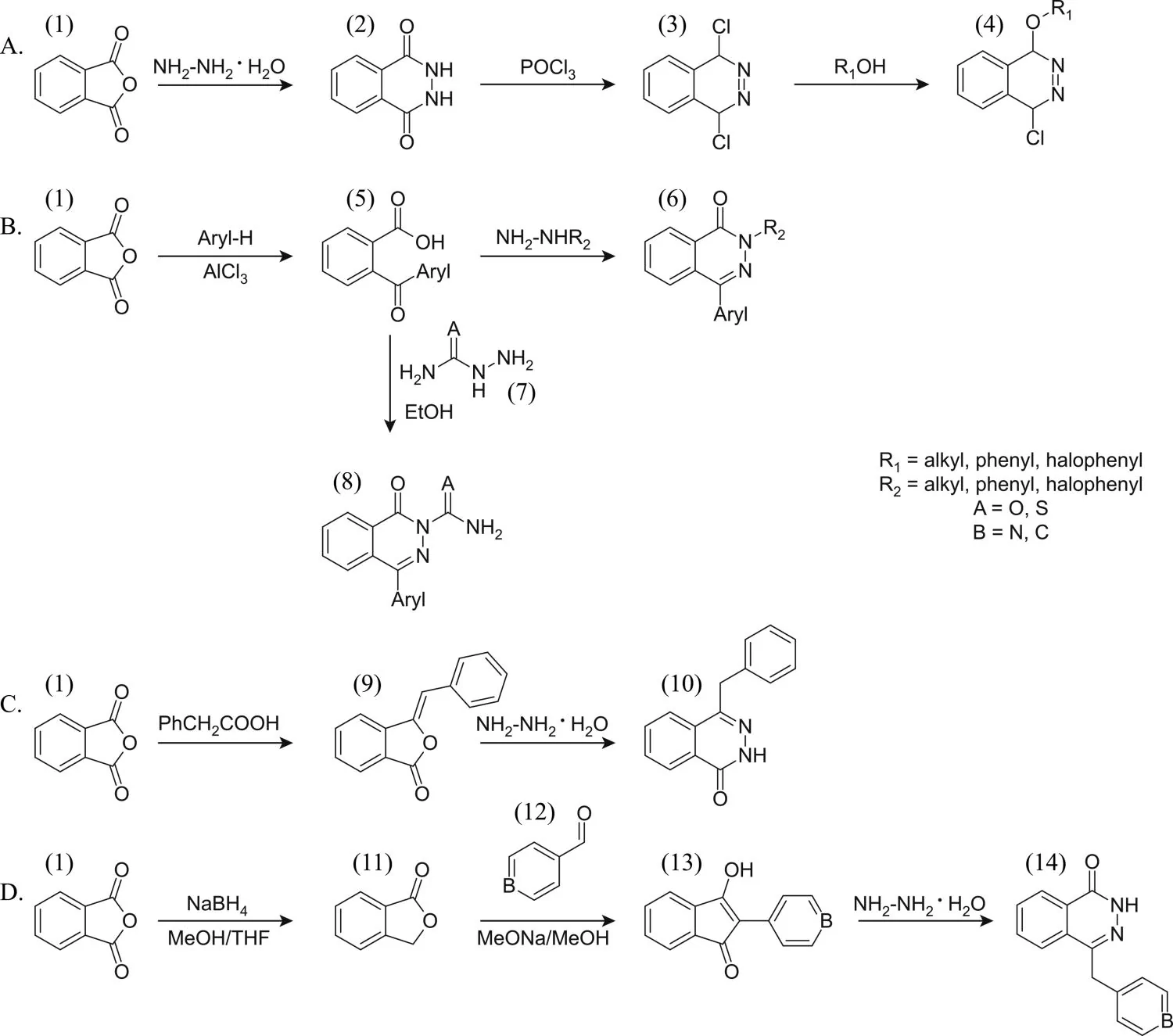
Phthalic anhydride as a starting compound for the synthesis of phthalazine derivatives. A: with hydrazine for preparation of 1,4-disubstituted phthalazine; B: with aryls or hydrazinecarboxamide for preparation of 2,4-disubstituted phthalazin-1(*2H*)-ones; C: with phenylacetic acid and hydrazine for the synthesis of 4-benzyl-2*H*-phthalazin-1-ones; D: *via* isobenzofuran-1(3*H*)-one for preparation of 4-substituted phthalazin-1(2H)-ones. Designed by the authors using Marvin Cloud. No copyrighted material was reused.

The processes with phthalic anhydride (1) are conducted in the presence of a solvent like EtOH or acetic acid yielding 2,3-dihydrophthalazine-1,4-dione (2). The ketone is usually further reacted with POCl_3_ to give 1,4-dichlorophthalazine (3). Chlorine analogue reacts with appropriate alkanol or substituted phenol in dimethyl formamide (DMF) to give different derivative (4) (Figure 7A)^37^^,^^93–95^. This method is commonly used to obtain an intermediate for the synthesis of more complex phthalazine-based compounds. Recently this protocol has been used for the preparation of novel triazolo-phthalazine hybrids as potential PCAF inhibitors^65^, DNA intercalators and DNA topoisomerase 2-alpha (TOP2A) inhibitors^67^, pyrazole phthalazine derivatives as new α-glucosidase inhibitors^96^. Carling et al. used 2,4-dichlorophthalazine obtained in this way for the preparation of phenyl-6–(2-pyridyl)methyloxy-1,2,4-triazolo[3,4-*a*]phthalazines with the great affinity for γ-aminobutyric acid-A benzodiazepine receptor^85^. Another application of this procedure is the synthesis of the anticancer agents with the potential antiangiogenic activity *via* VEGFR-2 inhibition^97^.

In another procedure with phthalic anhydride (1), the aromatic hydrocarbons were used as the starting reagents (Figure 7B). The reaction in the presence of AlCl_3_ gave an intermediate product, the acyl derivative of benzoic acid (5). The cyclisation reaction with hydrazine or substituted hydrazine afforded 2,4-disubstituted phthalazin-1(2*H*)-one (6). In the reaction of the acyl derivative (5) with hydrazinecarboxamide (7), 4-aryl-1-oxophthalazine-2(1*H*)-carboxamide (8) was obtained^94^.

Emam et al. used phthalic anhydride (1) and 2-phenylacetic acid to obtain 4-benzyl-2*H*-phthalazin-1-one (10) as an intermediate compound (Figure 7C). This approach was used for the preparation of various phthalazine derivatives that turned out to be selective agents against breast cancer cells and induced the EGFR-mediated apoptosis^63^.

An alternative approach to the synthesis of 4 arylmethylphthalazin-1(*2H*)-ones using phthalic anhydride (1) as the starting material was developed by Zhang et al.^98^ (Figure 7D). In this reaction, isobenzofuran-1(3*H*)-one (11) was obtained by reduction of the anhydride with NaBH_4_ in dry THF. Next, compound 11 was converted into 3-hydroxy-2-(pyridine–4-yl)-1*H*-inden-1-one (13) by treatment with 4-pyridinecarboxaldehyde (12), and the intermediate was subsequently reacted with hydrazine hydrate to give the key compound (14). The compounds were designed and prepared as potential anticancer agents.

**Figure 8.**
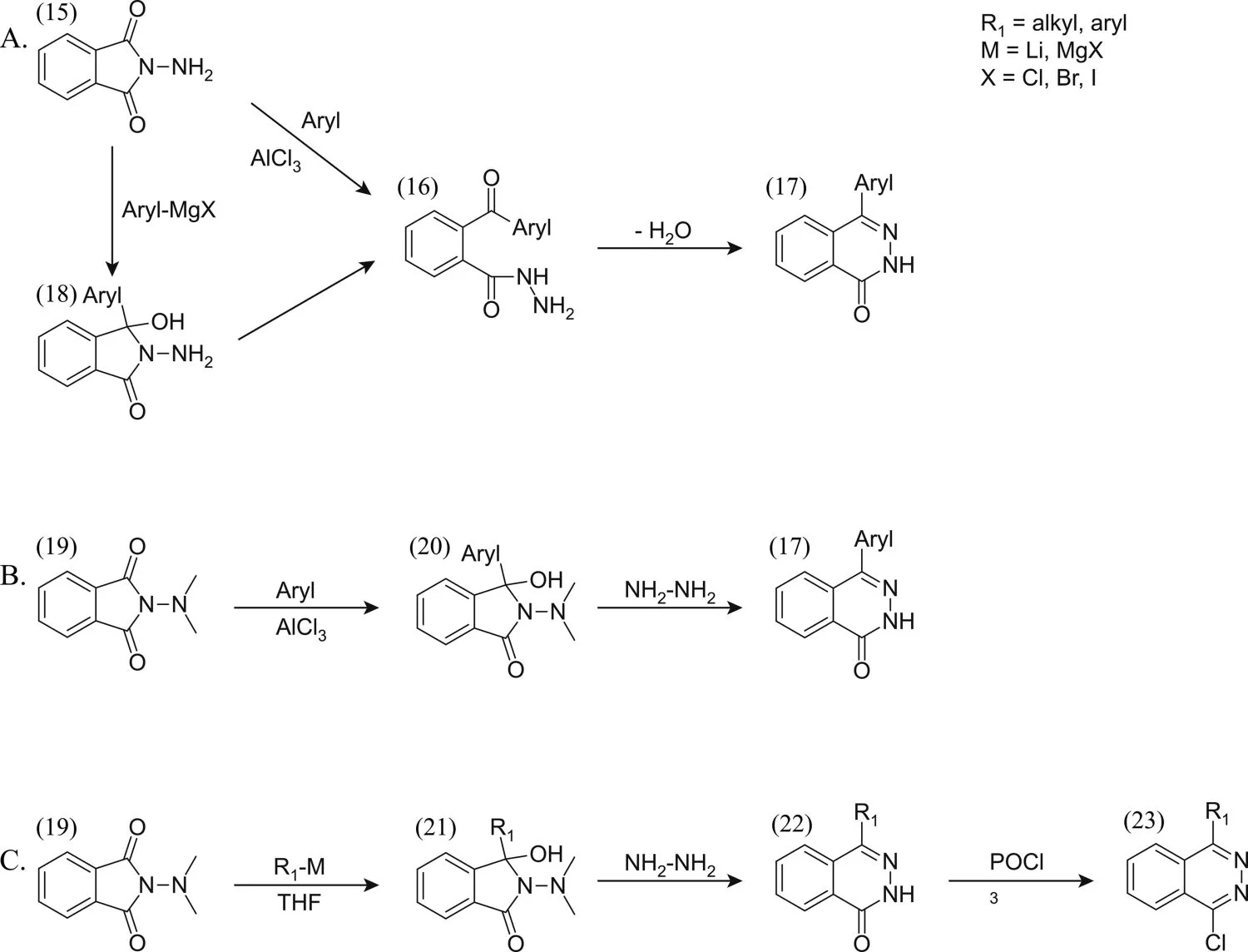
N-Aminophthalimide and its derivatives as starting compounds for the synthesis of phthalazines. A: with aryls *via* the AlCl_3_-catalysed Friedel–Crafts reaction or with the Grignard reagent (Aryl-MgX); B: N,N-dimethylaminophthalimide, aryls in the presence of AlCl_3_ and hydrazine; C: N,N-dimethylaminophthalimide with organometallic compounds (R-M) andhydrazine. Designed by the authors using Marvin Cloud. No copyrighted material was reused.

The other frequently used starting reagent is phthalimide (Figure 8). The synthesis procedure of 4-arylphthalazinones (17) based on the N-aminophthalimide (15), aryls and the Friedel–Crafts acylation reaction was reported by Ismail et al. The reaction proceeds through the 2-aroylbenzoic acid hydrazide (16), which undergo a cyclocondensation to afford the phthalazinone skeleton (17). This protocol provides the opportunity to obtain compounds with Ph or substituted Ph at the C-4 position with the 63–74% yield. The reaction with the Grignard reagent (Aryl-MgX) gives the same products (17) (Figure 8A)^99^. This procedure using N-aminophthalimide is imperfect due to formation of byproducts resulting from disubstitution or bisaddition reactions. The use of N,N-disubstituted N-amino phthalimide allows for selective monosubstitution or monoaddition. The steric hindrance present in the N,N-dialkyl derivatives was likely to prevent introduction of another substituent under the Friedel-Crafts reaction conditions^92^.

Saito et al. used N,N-dimethylaminophthalimide (19) as the starting reagent for the synthesis of phthalazine. In the reaction with aromatic hydrocarbons, enhanced by AlCl_3_ as a catalyst, 3-aryl-3-hydroxyisoindol-1-ones were obtained (20). Finally, after reacting with hydrazine, 4-substituted phthalazinones (17) were formed. Several aryl substituted derivatives were obtained with good yields **(**Figure 8B)^100^.

The three-step method using N-aminophthalimide (19) for the 4-substituted chlorophthalazines (23) preparation was proposed by Nguyen et al.^101^. The selective monoaddition of alkyl, aryl, and heteroaryl organometallic compounds to N,N-dimethylaminophthalimide (19) affords 3-substituted 3-hydroxyisoindolinones (21). Then, they were converted to chlorophthalazines (23) by the reaction with hydrazine, followed by chlorination with POCl_3_ (Figure 8C).

**Figure 9.**
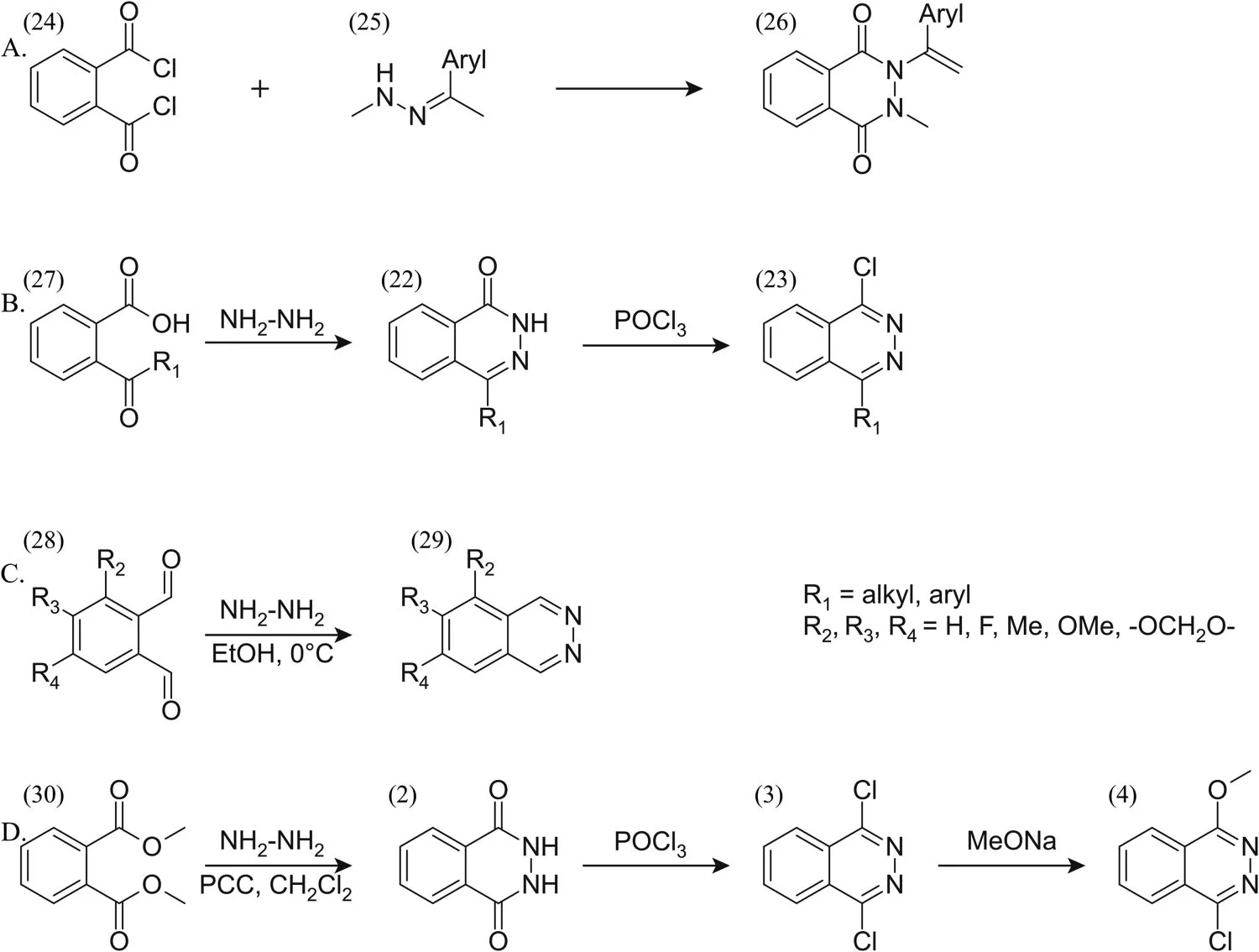
Phthalic acid derivatives or phthalaldehyde as starting compounds for synthesis of phthalazine derivatives. A: synthesis of 2-methyl-3–(1-arylvinyl)-2,3-dihydrophthalazine-1,4-dione derivatives using phthaloyl chloride; B: synthesis of 4-substituted phthalazin-1-ones from o-acylbenzoic acid; C: synthesis of 5,6,7-substitutes phthalazine from phthalaldehyde and hydrazine; D: synthesis of 2,3-dihydrophthalazine-1,4-diones (2) from o-diester. Designed by the authors using Marvin Cloud. No copyrighted material was reused.

Apart from the phthalimide core, the other 1,2-dicarbonyl compounds are also used in the synthesis of phthalazines. In the preparation of specifically 2,3-disubstituted phthalazine derivatives (26, 2-methyl-3–(1-arylvinyl)-2,3-dihydrophthalazine-1,4-dione) phthaloyl chloride (24) and N-methyl acetophenone hydrazones (25) were used as the starting reagents (Figure 9A)^94^. The cyclisation reaction of o-acylbenzoic acids (27) with hydrazines results in generation of 4-substituted phthalazin-1-ones (22) (Figure 9B). This is a direct and efficient method to obtain compounds having different substituents at the 4-position of the phthalazinone ring^92^^,^^94^. In this way there were obtained the anticancer agents with potential antiangiogenic activity resulting from VEGFR-2 inhibition^97^.

Shubin et al. used substituted hydrazines in the reaction with o-acylbenzoic acid. The cyclisation of 2-nitro-5-chloro phenylhydrazine with acyl benzoic acids gives 2–(2-nitro-5-chlorobenzene)-4-phthalazin-1-ones^102^. The other researchers used this process for preparation of phthalazine as a key unit for the benzo[4,5]imidazo[2,1-*a*]phthalazine preparation^102^^,^^103^. Mourad et al. used the reaction to obtain 4-arylphthalazin-1(2*H*)-ones as an intermediate reagent for synthesis of more complicated phthalazine-based compounds displaying an antimicrobial activity^104^.

Some phthalazine derivatives substituted in the aryl ring (29) were obtained in the reaction of phthalaldehyde (28) and hydrazine in EtOH as a reaction medium (Figure 9C)^36^. The process of dimethylphthalate (30) with hydrazine results in formation of 2,3-dihydrophthalazine-1,4-dione (2) which undergoes chlorination with POCl_3_ to give 1,4-dichlorophthalazine (3). The subsequent treatment with sodium methoxide afforded a methoxychloride derivative (4) (Figure 9D)^105^. This procedure was used for fused phthalazine-based compounds preparation.

### Monocarbonylaryl derivatives and other compounds as starting reagents

**Figure 10.**
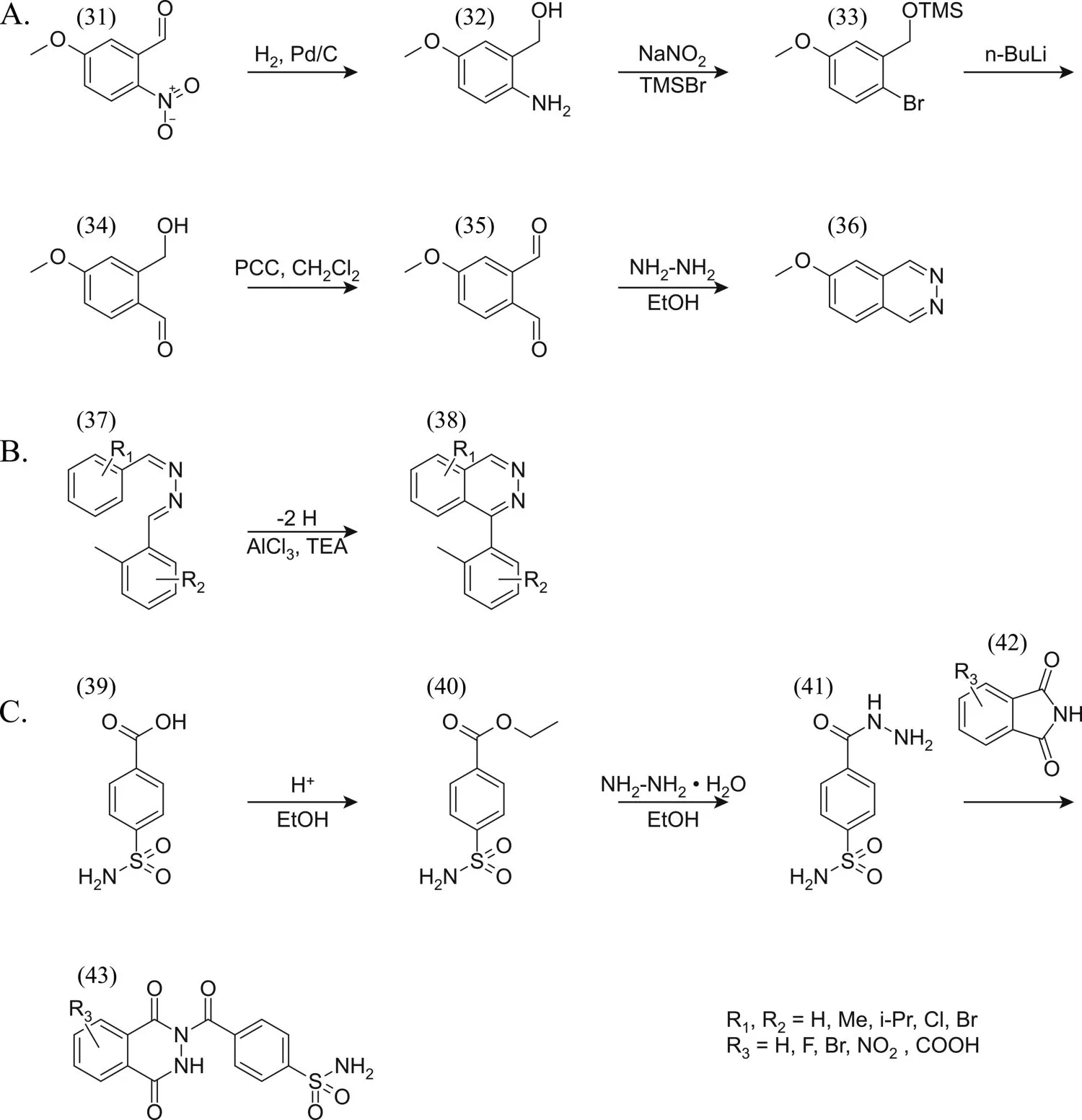
Monocarbonylaryl derivatives and aromatic aldazines as starting compounds for synthesis of phthalazine derivatives. A: multi-step synthesis of 6-methoxyphthalazine from 2-amino-5-methoxybenzaldehyde *via* aromatic 1,2-dialdehyde, B: synthesis of 1-aryl phthalazines from aldazines catalysed by Lewis acid. C: synthesis of 4–(1,4-dioxo-1,2,3,4-tetrahydrophthalazine-2-carbonyl) benzenesulfonamides from 4-sulfamoylbenzoic acid as the starting material. Designed by the authors using Marvin Cloud. No copyrighted material was reused.

Tsoungas and Searcey used the reaction of aromatic 1,2-dialdehyde and hydrazine for preparation of 6-methoxyphthalazine (33) (Figure 10A)^106^. They started with 2-methoxy-5-nitrobenzaldehyde (31), transformed to 2-methoxy-5-aminobenzyl alcohol (32) by the catalytic hydrogenation. Diazotation of amine, in the presence of trimethylsilyl halide, either chloride or bromide (TMSBr), gave a bromine derivative (33). The halogen lithium exchange was followed by the pyridinium chlorochromate (PCC) oxidation. Compound (36) was designed and obtained as a key precursor for the synthesis of phthalazine-based DNA intercalators.

Robev proposed the synthesis of phthalazine from aromatic aldazines (37) (Figure 10B). The Lewis acid AlCl_3_ together with triethylamine TEA as a base mediated formation of the target 1-arylphthalazines (38). The yields ranged from 15% to 70%^107^.

Another reaction sequence for the synthesis of phthalazine-based sulphonamides acting as CA and AChE inhibitors was developed by Türkeş et al. (Figure 10C). They used 4-sulfamoylbenzoic acid as the starting material (39). The phthalazinedione scaffold (43) was obtained from the reaction of 4-(hydrazinecarbonyl)benzenesulphonamide (41) with phthalimide (42)^90^.

### Synthesis reactions involving tetrazines

**Figure 11.**
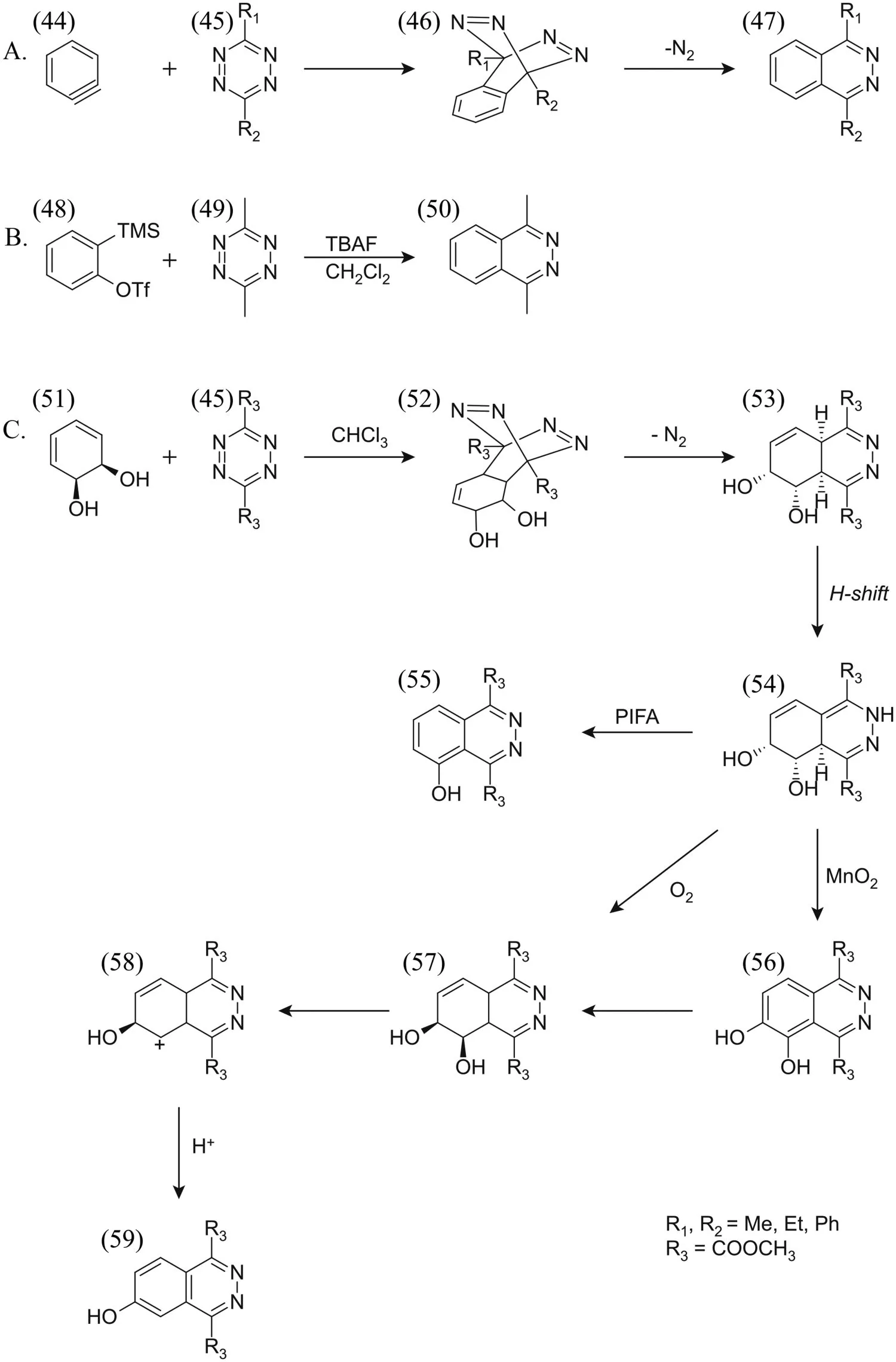
Phthalazine derivatives synthesis involving tetrazine. Synthesis of 2,4-disubstituted phthalazine A: with benzyne; B: with benzyne precursor: C: synthesis of hydroxyl derivatives of phthalazine from dimethyl-1,2,4,5-tetrazine-3,6-dicarboxylate and benzene cis-diol. Designed by the authors using Marvin Cloud. No copyrighted material was reused.

Phthalazine synthesis reactions using benzyne (44) and 1,2,4,5-tetrazine (45) as starting materials proceeding through the Diels–Alder/retro-Diels–Alder process are also applied. Such et al. developed a triple aryne–tetrazine reaction that proceeds as a one-pot reaction. This is a reaction that does not require a metal as a catalyst, proceeds very quickly (about 5 min) in air. Even though a broad substitution spectrum was not obtained, the authors declared a possibility of using this procedure for the synthesis of other analogues. The obtained compounds (47) were used for preparation of new fused phthalazine-based systems^108^^,^^109^. In these reactions, the benzyne precursor 2-(trimethylsilyl)phenyl trifluoromethanesulphonate (TMSOTf) was used (48), which, in the presence of tetra-N-butylammonium fluoride (TBAF) as a F^-^ source, gave the starting reagent (Figure 11A and 11B). Dimethyl, diethyl, and diphenyl derivatives were obtained (47, 50).

The inverse Diels–Alder reaction was examined for the construction of the phthalazine skeleton substituted by the hydroxyl group(s). As starting reagents, dimethyl 1,2,4,5-tetrazine-3,6-dicarboxylate (45) and benzene cis-diol (51) as a dienophile in CHCl_3_ were used^110^. After the nitrogen extrusion, the initially formed tricyclic intermediate (52) was transformed into a dihydrodiol containing a 1,4-dihydropyridazine ring (53). The phthalazine-type dihydrodiol is unstable and undergoes aromatisation readily. Oxidation of this intermediate product using various oxidants (O_2_, phenyliodo-bis(trifluoroacetate) (PIFA), and MnO_2_) resulted in the formation of 5-hydroxy- (55), 6-hydroxy- (59) and 5,6-dihydroxy- (56) phthalazine derivatives. The desired phthalazine derivatives were obtained in good yields (Figure 11C).

### Synthesis from heterocyclic compounds

**Figure 12.**
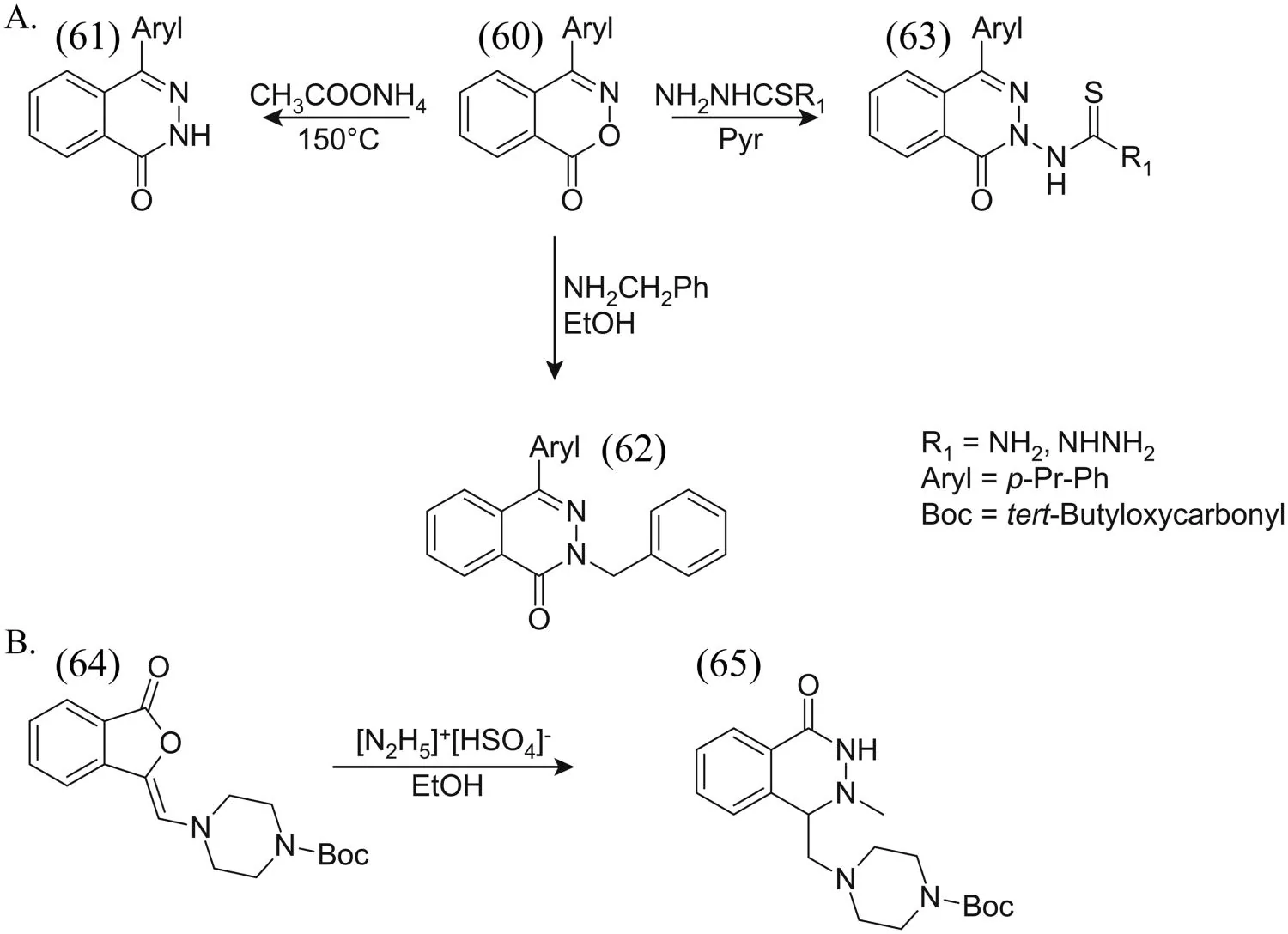
Phthalazine synthesis based on heterocyclic compounds. A: Synthesis of 4-substituted phthalazin-1(2*H*)-ones and 2,4-disubstituted phthalazin-1(2*H*)-ones from 4-aryl-1*H*-benzo[*d*][1,2]oxazin-1-one; B [N_2_H_5_]^+^[HSO_4_]^-^- catalysed synthesis of 3,4-dihydrophthalazin-1(2*H*)-one-piperazine hybrids from isobenzofuran-1(3*H*)-one. Designed by the authors using Marvin Cloud. No copyrighted material was reused.

Mourad et al. obtained phthalazine derivatives with the antimicrobial activity by means of transformation reaction of another heterocyclic ring^104^. The reaction of 4-aryl-1*H*-benzo[*d]*[1,2]oxazin-1-one (60) in the presence of CH_3_COONH_4_ at 150 °C gave 4-arylphthalazin-1(*2H*)-one (61), while with PhCH_2_NH_2_ in EtOH gave the 2-benzyl derivative (62). If H_2_NHCSX in pyridine was used, 2- thiourea derivatives (63) were obtained (Figure 12A).

Another heterocyclic system used for the synthesis of phthalazine derivatives is 3-oxoisobenzofuran-1(3*H*)-ylidene (Figure 12B). (*Z*)-*tert*-butyl-4-((3-oxoisobenzofuran-1(*3H*)-ylidene)methyl)piperazine-1-carboxylate (64) as a starting reagent was converted into corresponding phthalazine − piperazine conjugate (65) by treatment with hydrazine sulphate in EtOH. Then *tert*-butyl 4-((4-oxo-3,4-dihydrophthalazin-1-yl)methyl)piperazine-1-carboxylate (65) was applied as a key intermediate for the preparation of phthalazine-piperazine-pyrazole conjugates with anticancer activity^111^.

### Multicomponent reactions

In recent years, multicomponent reactions (MCRs) have emerged as a powerful and versatile strategy in the synthesis of structurally diverse organic molecules. This one-step process enables rapid and efficient preparation of compound libraries. It involves at least three or four reactants that combine to form a single desired product incorporating structural elements from all starting materials. The reaction proceeds through a network of equilibria that ultimately converge in an irreversible step leading to product formation. The efficiency and selectivity of MCRs are largely influenced by various factors, including the solvent, catalyst, concentration, temperature as well as the nature and functional groups of the starting materials. Within the pharmaceutical sciences, MCRs play a crucial role in the synthesis of low molecular weight, drug-like compounds. They facilitate rapid exploration of chemical space and accelerate early-stage drug discovery^112^^,^^113^.

**Figure 13.**
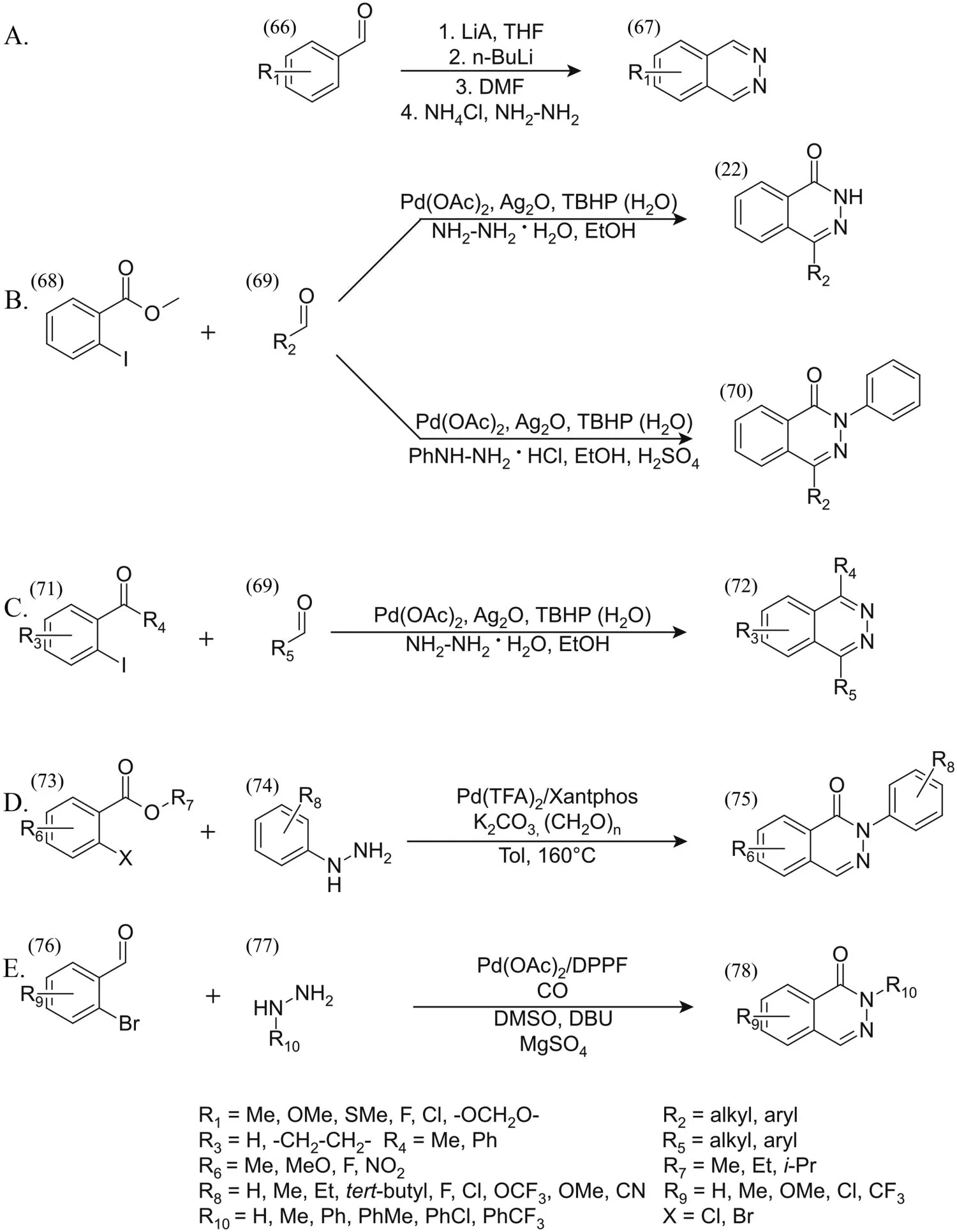
Phthalazine synthesis utilising multicomponent reactions (Part I). A: synthesis of variously substituted phthalazines in the aryl moiety from benzaldehydes and hydrazine in the presence of lithium amide (LiA); B: synthesis of 4-substituted and 2,4-disubstituted phthalazin-1(2*H*)-ones from methyl 2-iodobenzoate, aldehyde and hydrazine affected by palladium acetate (Pd(OAc)_2_) and Ag_2_O; C: synthesis of 1,4-disubstituted phthalazines from 2-iodo aromatic ketones affected by Pd(OAc)_2_)/Ag_2_O; D: Pd-catalysed synthesis of 2-substituted phthalazin-1(2*H*)-ones from 2-halobenzoic acid esters with paraformaldehyde as carbon source, XantPhos - 4,5-bis(diphenylphosphino)-9,9-dimethylxanthene; E: Pd(OAc)_2_-catalysed synthesis of 2-substituted phthalazin-1(*2H*)-ones from *o*-halobenzaldehyde, hydrazine and CO. Designed by the authors using Marvin Cloud. No copyrighted material was reused.

A universal method of phthalazines synthesis (67) was elaborated by Kessler and Wegner. It is the one-pot strategy that allows to obtain 4- to 8-substituted phthalazines from simple aromatic aldehydes (66) with good to excellent yields (Figure 13A). The conversion of benzaldehydes into directed *ortho*-metalated groups (DMG) by lithium amide (LiA) is a fundamental step in this approach^91^^,^^105^. Although aldehydes themselves are not directed groups, they can react with lithium amide to form R-amino alcohol salts, which then generate moderate to good DMG. Finally, the phthalazine scaffold is formed. The authors applied N,N,N-trimethylethylenediamine (TMEDA) and lithium amide (LiA1) in order to form a R-aminoalkoxide. A new reagent, bis(2-methoxyethyl)amine (BMEA) may also be used for this purpose constituting a much cheaper solution than TMEDA. The method facilitates synthesis involving multiple electron-withdrawing as well as electron-donating substituents.

Suchand and Satyanarayana reported the Pd-catalysed cyclocondensation of an aromatic ester (68) with routinely applied aldehydes (69) as a source of a carbon atom for formation of the phthalazine ring (22) (Figure 13B)^114^. The synthesis process involves the acylation reaction of methyl 2-iodobenzoate (68) by aldehyde (69) affected by palladium acetate (Pd(OAc)_2_), silver oxide and TBHP (tertbutyl hydroperoxide) in water, followed by treatment with hydrazine hydrate to yield 4-substituted phthalazin-1(*2H*)-one (22). Ethanol is the best solvent for the synthesis of the compounds and the method gives satisfactory yields. Using aryl substituted hydrazines (ArNHNH_2_) as the starting reagent and slightly changing the reaction conditions, e.g. additionally using H_2_SO_4_, the same authors obtained several 2-aryl-4-substituted phthalazin-1(2*H*)-ones (70) (Figure 13B).

In the analogous process introduced by other authors, 1-phenylethan-1-one and other ketones were used under similar synthetic conditions. Using this protocol, they prepared several analogues of the 1,4-disubstituted (alkyl, aryl) phthalazine group with a yield of about 60%^34^. In the other process, the authors used 2-iodophenylketones (71) and the same synthesis conditions^114^. Using this procedure, they obtained several analogues of the 1,4-disubstituted (alkyl, aryl) phthalazine group (72) with a yield of 60% (Figure 13C).

Another useful and convenient carbon source for MCRs is paraformaldehyde. This is a cheap and relatively safe reagent. A one-pot method of phthalazine synthesis using paraformaldehyde as a carbon source was developed by Wang et al. This is a Pd-catalysed three-component process using a 2-halobenzoic acid ester (73) and an arylhydrazine (74) as the principal reactants. This synthetic approach employs Pd(TFA)_2_ and Xantphos (4,5-bis(diphenylphosphino)-9,9-dimethylxanthene) as catalysts, K_2_CO_3_ as the reaction base, and toluene as the reaction medium. Several derivatives with modifications in both aromatic rings (75) were obtained (Figure 13D)^115^.

The palladium-catalysed reaction leading to phthalazine derivatives preparation (78) was also elaborated by Wu et al. ortho-halobenzaldehydes (76) and hydrazine monohydrate derivatives (77) were used as base reagents and Pd(OAc)_2_ as a catalyst. CO was the carbon source in this process. The method makes it possible to use a wide range of reagents, and the target compounds are obtained with satisfactory yields (Figure 13E)^116^.

**Figure 14.**
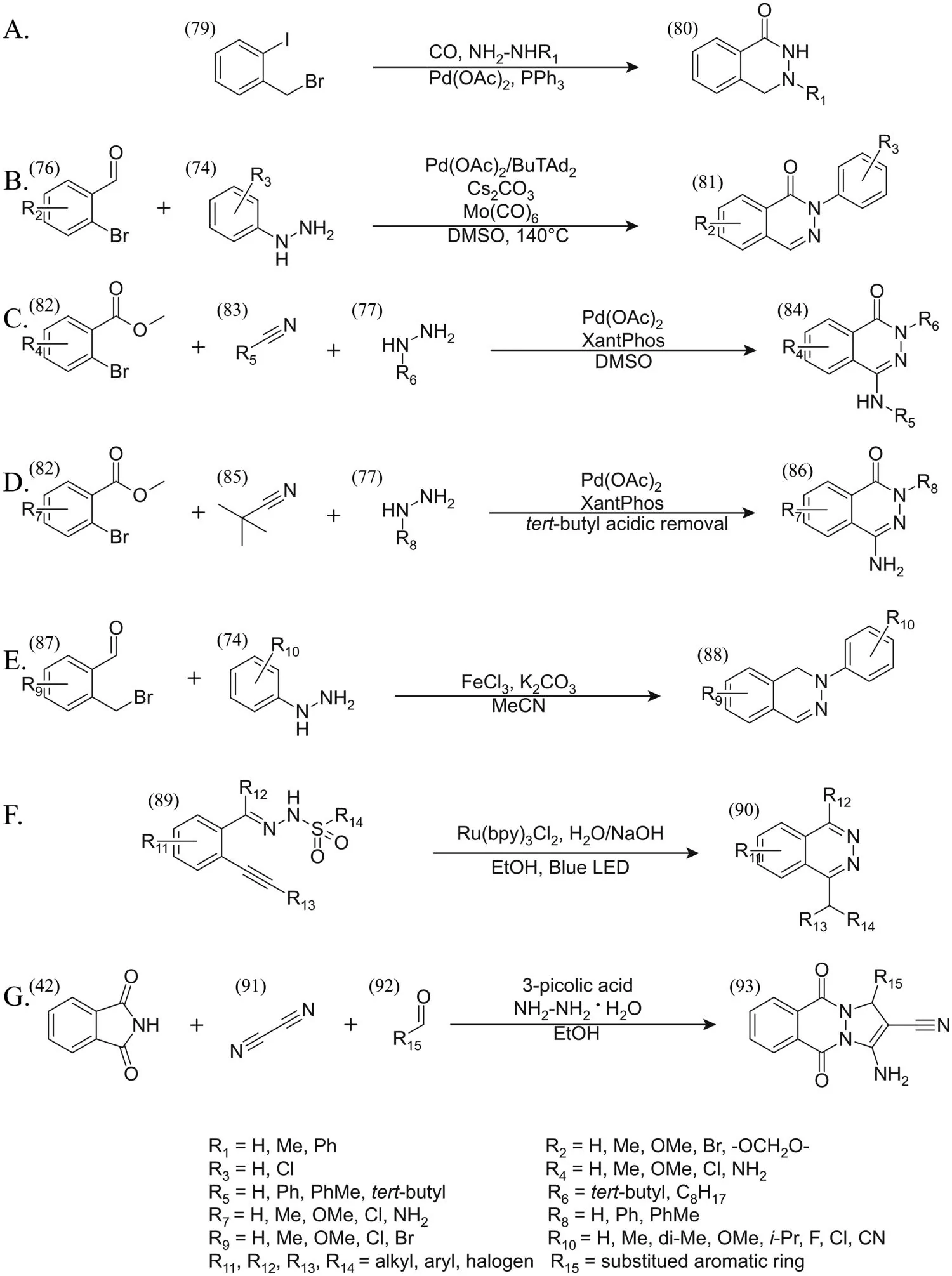
Phthalazine synthesis utilising MCRs (Part II). A: Pd-catalysed synthesis of 3-substituted 1,2,3,4-tetrahydrophthalazine-1-one derivatives from 2-iodobenzyl bromide, hydrazine and CO; B: microwave-promoted Pd-catalysed synthesis of 2-arylphthalazin-1(2*H*)-ones from 2-bromobenzaldehyde and hydrazine using Mo(CO)_6_; C, D: Pd-catalysed synthesis of 2-substituted 4-aminophthalazin-1(2*H*)-ones from o-halobenzoate, hydrazine and isocyanide; E: FeCl_3_-catalysed synthesis of 2-aryl-1,2-dihydrophthalazines from 2-bromomethylbenzaldehyde and arylhydrazine; F: the photocatalyzed synthesis of trisubstituted phthalazines from N′-(2-(phenyl ethynyl)benzylidene)benzenesulfonohydrazone; G: 1-substituted 3-amino-5,10-dioxo-5,10-dihydro-1*H*-pyrazolo[1,2-b]phthalazine-2-carbonitrile. Designed by the authors using Marvin Cloud. No copyrighted material was reused.

2-Iodobenzyl bromide (79) as a bifunctional reagent instead of the carbonyl derivative was also used in preparation of phthalazines. It was transformed *via* a palladium-catalysed intramolecular hydrazinocarbonylation to 1,2,3,4-tetrahydrophthalazine-1-one derivative (80), which allowed some products to be obtained with yields as high as 85% (Figure 14A). Reactions with methylhydrazine were characterised by great selectivity, as only a few derivatives of this type were obtained^117^.

Another method has been developed that avoids the use of free toxic CO, even though a complex Mo(CO)_6_, is applied as a precursor of CO. Similarly to the method described above, ortho-bromoarylaldehydes (76) and arylhydrazines (74) are used in the reaction as starting reagents (Figure 14B). The process is palladium-catalysed and enhanced by microwave radiation, which shortens the reaction time. In this way, a series of 2-arylphthalazin-1(2*H*)-ones (81) modified in both aromatic rings were obtained in satisfactory yields (40–67%). This approach is simple, efficient, and environmentally friendly. The reaction can also be conducted on a small scale^118^.

The one-pot palladium-catalysed multicomponent synthesis of 4-aminophthalazin-1(2*H*)-ones (84) with substituted o-halobenzoate (82) and isocyanides (83) was elaborated by Vlaar and al. (Figure 14C and 14D)^119^^,^^120^. The reaction involves isocyanide (83) and free hydrazine, XantPhos as the ligand as well as one of the few palladium catalysts. This is a unique method that allows the regioselective introduction of various substituents onto the C5–C8 positions of the aryl moiety. The target compounds (84) were obtained with high yields up to 99% for some compounds^120^. In the other study, the same team employed a palladium-catalysed MCR involving isocyanide insertion, mainly using *ter*t-butyl isocyanide. They obtained a series of compounds bearing a *tert-*amino substituent at position 4 (84). They also developed a method for preparing compounds with a free amine group at position 4 of the phthalazine ring (86) with very high yields (up to 99%). Subsequently they were subjected to further transformations^119^.

The synthesis of 2-aryl-1,2-dihydrophthalazines (88) from 2-(bromomethyl)benzaldehydes (87) and arylhydrazines (74) was developed by Aljaar et al. The reaction was conducted in MeCN at 100 °C using FeCl_3_ as a catalyst and K_2_CO_3_ as a base. The transformation of the compounds is considered to proceed as an intermolecular condensation/intramolecular nucleophilic substitution. There were obtained a dozen or so compounds with the yields ranging from 60% to 91% (Figure 14E). This method provides a simple and efficient approach to the synthesis of 2-aryl modified 1,2-dihydrophthalazines^121^.

Brachet et al. developed a synthesis reaction of trisubstituted phthalazines (90) that follows the principles of green chemistry (Figure 14F)^122^. A broad range of readily accessible N′-(2 (phenyl ethynyl) benzylidene)benzenesulphonohydrazones (89) was used as precursors. The process involves a new cascade reaction, initiated by visible light photocatalysis. In this reaction a radical hydroamination reaction was followed by a radical Smiles rearrangement. Tris(bipyridine)ruthenium(II) (Ru(bpy)_3_Cl_2_·6H_2_O) was used as the photocatalyst. More than 20 derivatives of this type (90) were obtained with good yields, and this reaction offers the possibility to obtain a wide spectrum of analogs.

Recently, a commonly used substrate phthalimide (42), has been used for preparation of nitrilo-phthalazine derivatives (93) in the one-pot procedure (Figure 14G). Compounds were formed in the four-component condensation reaction of phthalimide, hydrazine hydrate, aromatic aldehyde and malononitrile (84) in EtOH with 3-picolinic acid^123^.

**Figure 15.**
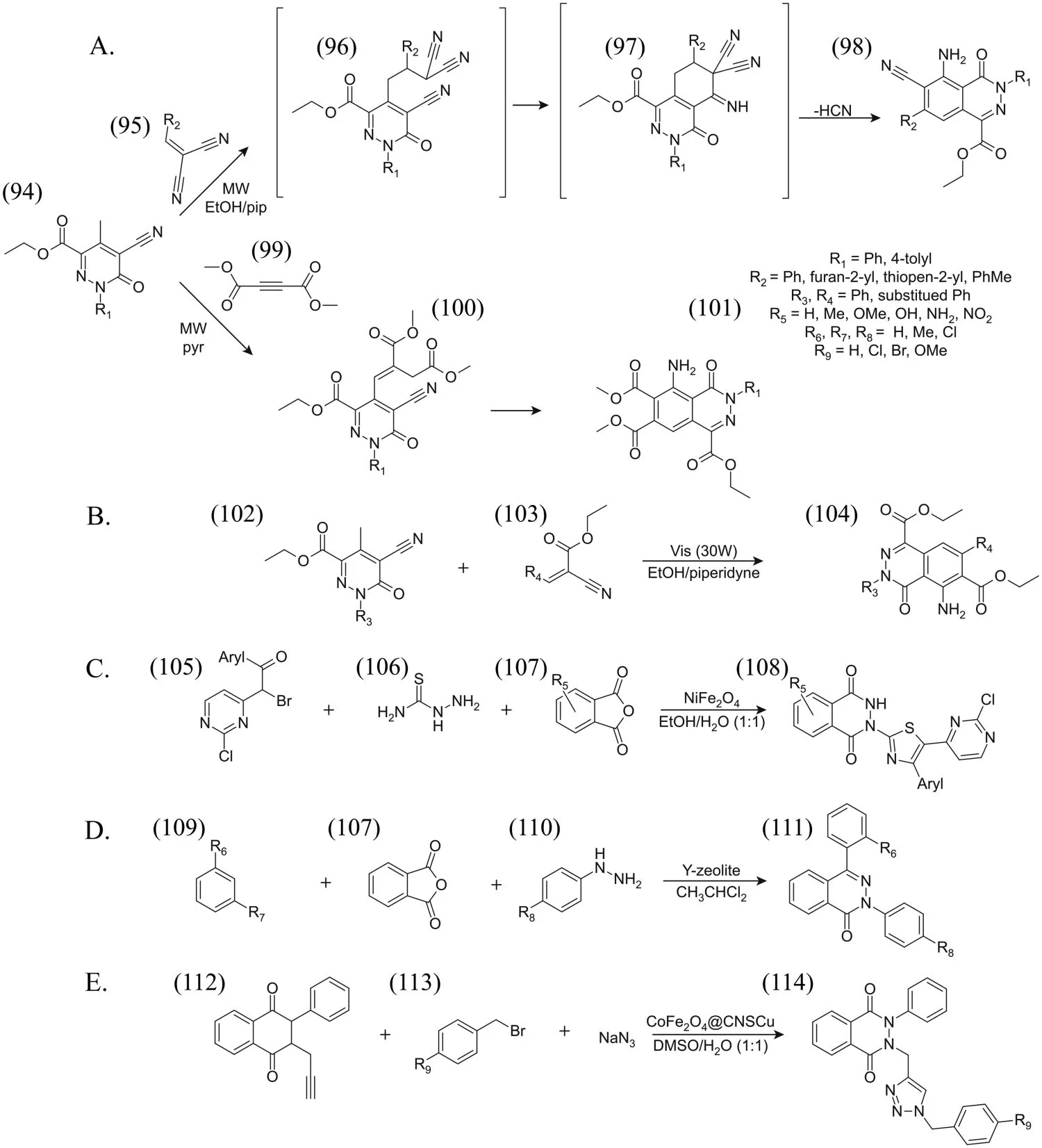
Phthalazine derivatives synthesis utilising MCRs (Part III). A: synthesis of polyfunctionally substituted phthalazin-1(2H)-ones based on 3-oxo-2,3-dihydropyridazine-4-carbonitrile with the assistance of microwave heating; B: with the assistance of visible-light; C: NiFe_2_O_4_-catalysed synthesis of 2,3-dihydrophthalazine-1,4-dione derivatives containing pyrimidin-4-ylthiazol-4-yl unit; D: zeolite-catalysed synthesis of 2,4 diphenylphthalazin-1(2*H*)-ones ; E:. CoFe_2_O_4_@CNSCu-nanocatalysed synthesis of triazole-based hybrids containing 2-phenyl-2,3-dihydro-phthalazine-1,4-dione unit from 2,3-dihydronaphthalene-1,4-dione and sodium azide. Designed by the authors using Marvin Cloud. No copyrighted material was reused.

Nazmy et al. developed the synthesis reaction of polyfunctionally substituted phthalazines (98, 101) from 3-oxo-2,3-dihydropyridazine-4-carbonitrile (94) as a key reagent (Figure 15A). The one-pot reaction included ethyl 1-aryl-5-cyano-1,6-dihydro-4-methyl-6-oxo-3-pyridazine-carboxylate (94) and cinnamonitrile derivative (95). These reagents formed a 1:1 Michael adduct (96), which subsequently underwent cyclisation (97). This intermediate underwent aromatisation, eliminating HCN and finally yielding a phthalazine derivative (98). Similarly, in the reaction with acetylenedicarboxylate (99) in pyridine, the corresponding phthalazine was formed (101). This reaction also proceeds through a Michael adduct as an intermediate (100). The reaction was conducted in the presence of microwave heating with large yields^124^.

Recently, a visible-light-mediated approach, used as a green metal-free and efficient technique, was developed for the preparation of polyfunctionally substituted phthalazin-1(2*H*)-one derivatives (104) (Figure 15B). The reaction of pyridazine (102) with arylidene (103) was conducted in EtOH in the presence of a catalytic amount of piperidine under white LED lamp irradiation. The compounds were obtained in excellent yields of about 90%. Subsequent computational analysis indicated the antimicrobial potential of these compounds^125^.

Using a green, one-pot MCRs, Gudala et al. obtained phthalazine-based benzenesulfonamides containing a pyrimidin-4-ylthiazol-4-yl (108) unit, which exhibit anticancer activity (Figure 15C)^126^. NiFe_2_O_4_ nanoparticles served as an efficient and reusable heterogeneous catalyst. The synthetic protocol involved the reaction of an α-halo carbonyl compound (105) with thiosemicarbazide (106) and various phthalic anhydrides (107), leading to the formation of phthalazine and thiazole rings. In this way, substituted 2,6-dichloro-N-(2-chloro-3–(5-(2-chloropyrimidin-4-yl)-2–(1,4-dioxo-3,4-dihydrophthalazin-2(1*H*)-yl)thiazol-4-yl)phenyl)benzenesulfonamides (108) were obtained in the large yield.

Another multicomponent strategy leading to 2,4-diphenylphthalazin-1(2*H*)-ones (111) involved the use of arenes (109), phthalic anhydride (107) and phenylhydrazines (110) (Figure 15D). The reaction was conducted in the presence of the efficient, recyclable heterogeneous catalyst HY-zeolite (crystalline aluminosilicate zeolite Y), providing high yields in short reaction times. The obtained compounds exhibited anticancer activity^127^.

The synthesis of novel triazole-based hybrids containing the 2-phenyl-2,3-dihydro-phthalazine-1,4-dione moiety (114) was conducted using a three-component CoFe_2_O_4_@CNSCu-nanocatalysed reaction (Figure 15E). 4-Substituted (bromomethyl)benzene, 2-phenyl-3-(prop-2-ynyl)-2,3-dihydronaphthalene-1,4-dione (112) and sodium azide (NaN_3_) were used as substrates. Various alkyl halides, including electron-withdrawing and electron-donating substituents, as well as sterically demanding groups, are tolerated by the approach. Preliminary biological evaluation revealed that the obtained compounds inhibited PARP-1, indicating their potential anticancer^128^.

### Future directions

The synthesis of the phthalazine core is well established and supported by numerous reliable approaches. A notable degree of chemical complexity in constructing this small molecule has been achieved using diverse, efficient, and reliable methodologies. The choice of the method is influenced by both the desired substitution pattern and the availability of starting reagents. Nevertheless, some dominant trends in the synthesis of phthalazines can be distinguished.

MCRs synthesis conducted as one-pot syntheses have been widely used in preparation of phthalazines in recent years^34^^,^^115^. They often employ non-standard reagents and utilise various carbon sources for phthalazine ring formation^117–119^^,^^121^^,^^124^. There is a tendency to use reagents that are readily commercially available, have a high degree of diversity and are non‑toxic. Some reactions are supported by UV radiation, which significantly shortens the reaction time. Various photocatalysts such as Ru(bpy)_3_(PF_6_)_2_, Ir(ppy)_3_ and Ru(bpy)_3_Cl_2_·6H_2_O were employed to improve the yield of the phthalazine products^118^^,^^122^. Additionally, a high level of functional‑group tolerance in the substrates has been observed^91^.

In organic synthesis, it is essential to adhere to the principles of green chemistry^122^. In this way synthetic procedures are designed to maximise the incorporation of substrates into the final product, the phthalazine ring. It is recommended to reduce energy consumption and to conduct reactions without hazardous solvents. Such approaches increase the selectivity and yield of the process, thereby improving reaction efficiency and reducing its adverse impact on the environment^36^^,^^124^.

Considering the structure of the compounds, the preparation of multifunctional substituted phthalazines appears to be of significant importance^124^. They offer great opportunities for modifying the initial structure and for designing and synthesising diverse phthalazine‑based derivatives.

Most of the reported compounds possess chlorine or methoxy substituents at position 1 and/or 4 of phthalazine ring. The approaches for obtaining amine derivatives, which involve the formation of a new C–N bond, have also been described^119^^,^^120^. These derivatives offer new possibilities for the synthesis of more complex phthalazine-based frameworks.

Despite the many advantages of one-step reactions, it appears that classical, multi-step synthesis will continue to be used^90106^. Currently, potential drugs are often designed with a specific molecular target in mind. This requires the presence of specific reactive groups positioned at defined sites on the phthalazine ring, and individual strategies will be necessary.

## Conclusions

Recent developments have significantly advanced our understanding of chemistry of phthalazine derivatives and their biomedical applications. The design of numerous new derivatives has increased the structural diversity of this class of compounds. Analysis of the influence of structural modifications on biological activity and molecular docking studies have allowed to delineate mechanisms of action of particular derivatives and in some cases even identify the molecular targets. The wide range of diverse, yet well-described, synthetic strategies encourage further work on phthalazine derivatives. However, it should be emphasised, that the majority of these compounds have been tested exclusively in *in vitro* settings, whereas their activity in more clinically relevant models remains unknown. Additionally, the findings obtained using computational methods should be verified using orthogonal validation strategies. Broad reactivity of these compounds constitute a significant obstacle in the development of phthalazine-based drugs. Therefore, thorough SAR-assisted modifications and toxicity evaluation should improve the compound’s efficacy and selectivity, thereby decreasing the potential side-effects of the phthalazine-based drug-candidates.

## Data Availability

Data sharing is not applicable to this article as no new data were created or analysed in this study.
